# Diatoms exhibit dynamic chloroplast calcium signals in response to high light and oxidative stress

**DOI:** 10.1093/plphys/kiae591

**Published:** 2024-11-09

**Authors:** Serena Flori, Jack Dickenson, Trupti Gaikwad, Isobel Cole, Nicholas Smirnoff, Katherine E Helliwell, Colin Brownlee, Glen L Wheeler

**Affiliations:** The Marine Biological Association, The Laboratory, Plymouth PL1 2PB, UK; The Marine Biological Association, The Laboratory, Plymouth PL1 2PB, UK; The Marine Biological Association, The Laboratory, Plymouth PL1 2PB, UK; The Marine Biological Association, The Laboratory, Plymouth PL1 2PB, UK; Biosciences, College of Life and Environmental Sciences, University of Exeter, Exeter EX4 4QD, UK; Biosciences, College of Life and Environmental Sciences, University of Exeter, Exeter EX4 4QD, UK; The Marine Biological Association, The Laboratory, Plymouth PL1 2PB, UK; Biosciences, College of Life and Environmental Sciences, University of Exeter, Exeter EX4 4QD, UK; The Marine Biological Association, The Laboratory, Plymouth PL1 2PB, UK; The Marine Biological Association, The Laboratory, Plymouth PL1 2PB, UK

## Abstract

Diatoms are a group of silicified algae that play a major role in marine and freshwater ecosystems. Diatom chloroplasts were acquired by secondary endosymbiosis and exhibit important structural and functional differences from the primary plastids of land plants and green algae. Many functions of primary plastids, including photoacclimation and inorganic carbon acquisition, are regulated by calcium-dependent signaling processes. Calcium signaling has also been implicated in the photoprotective responses of diatoms; however, the nature of calcium elevations in diatom chloroplasts and their wider role in cell signaling remains unknown. Using genetically encoded calcium indicators, we find that the diatom *Phaeodactylum tricornutum* exhibits dynamic calcium elevations within the chloroplast stroma. Stromal calcium ([Ca^2+^]_str_) acts independently from the cytosol and is not elevated by stimuli that induce large cytosolic calcium ([Ca^2+^]_cyt_) elevations. In contrast, high light and exogenous hydrogen peroxide (H_2_O_2_) induce large, sustained [Ca^2+^]_str_ elevations that are not replicated in the cytosol. Measurements using the fluorescent H_2_O_2_ sensor roGFP2-Oxidant Receptor Peroxidase 1 (Orp1) indicate that [Ca^2+^]_str_ elevations induced by these stimuli correspond to the accumulation of H_2_O_2_ in the chloroplast. [Ca^2+^]_str_ elevations were also induced by adding methyl viologen, which generates superoxide within the chloroplast, and by treatments that disrupt nonphotochemical quenching (NPQ). The findings indicate that diatoms generate specific [Ca^2+^]_str_ elevations in response to high light and oxidative stress that likely modulate the activity of calcium-sensitive components in photoprotection and other regulatory pathways.

## Teaser

Diatoms show elevated chloroplast calcium levels following exposure to high light and oxidative stress that correspond with the accumulation of hydrogen peroxide in the chloroplast.

## Introduction

Diatoms are unicellular stramenopile algae that are characterized by their production of a silica cell wall (frustule). They represent one of the most abundant photosynthetic organisms on our planet, playing a major role in both freshwater and marine ecosystems, and are particularly abundant in polar regions and in coastal upwelling systems, which experience substantial changes in light, temperature, and nutrient availability. Diatoms, therefore, require sophisticated cellular signaling mechanisms to sense and respond to their dynamic environment. Recent studies have identified cytosolic calcium signaling mechanisms that mediate the response of diatoms to stresses imposed by changes in salinity, temperature, and nutrients (Falciatore et al. 2000; Helliwell et al. 2021a, 2021b; Kleiner et al. 2022). However, the role of calcium signaling within diatom organelles remains unexplored.

In land plants (embryophytes), the chloroplast plays an important role in cellular calcium signaling, either through the modulation of cytosolic calcium ([Ca^2+^]_cyt_) elevations or through changes in Ca^2+^ within the chloroplast itself (Costa et al. 2018). Ca^2+^ concentrations in the thylakoid lumen are 3- to 5-fold higher than the stroma (Sello et al. 2018). The release of Ca^2+^ from the thylakoid lumen, or its entry across the chloroplast membrane, can result in substantial stromal Ca^2+^ elevations ([Ca^2+^]_str_). [Ca^2+^]_str_ exhibits a strong diel oscillation, increasing during the dark phase (Sai and Johnson 2002). As many enzymes within the Calvin–Benson–Bassham (CBB) cycle are inhibited by elevated Ca^2+^ (Charles and Halliwell 1980; Kreimer et al. 1988), it was proposed that the rise in [Ca^2+^]_str_ plays a direct role in the regulation of photosynthesis. Ca^2+^ is also implicated in the regulation of many other aspects of photosynthesis, suggesting it plays a central role in coordinating signaling pathways within the chloroplast (Hochmal et al. 2016). Several lines of evidence also point to an important role for chloroplast Ca^2+^ signaling in inorganic carbon acquisition and photoprotection in green algae (Petroutsos et al. 2011; Wang et al. 2016).

Although the importance of chloroplast Ca^2+^ signaling in plants and algae is becoming clear, the dynamics of Ca^2+^ within this organelle remain understudied in comparison with the cytosol. Studies using the bioluminescent calcium reporter aequorin have demonstrated that stimuli that induce [Ca^2+^]_cyt_ elevations, such as oxidative stress and salt (0.3 m NaCl) or the bacterial elicitor flg22 also induce [Ca^2+^]_str_ elevations, although the kinetics and amplitude of the Ca^2+^ elevations differ between the compartments (Manzoor et al. 2012; Nomura et al. 2012; Sello et al. 2016). Specific [Ca^2+^]_str_ elevations have been observed in *Arabidopsis* seedlings exposed to high temperature (Lenzoni and Knight 2019). These aequorin-based studies have revealed the presence of [Ca^2+^]_str_ elevations in response to many different stimuli, although they predominately report the response of a population of cells, which can obscure the complexities of Ca^2+^ dynamics within individual cells.

Direct imaging of Ca^2+^ dynamics in chloroplasts can be achieved by targeted expression of fluorescent genetically encoded calcium indicators (GECIs), although their use must be carefully controlled to avoid physiological impacts from the excitation light and fluctuations in stromal pH (Loro et al. 2016; Grenzi et al. 2021; Ugalde et al. 2021). Most fluorescent calcium reporters require excitation with light in the visible range, which has the potential to stimulate light-driven cellular responses, including photosynthesis itself. This problem has been overcome by imaging cells with intermittent light (e.g. every 5 s) to greatly reduce photosynthetic activity, although at the trade-off of limiting temporal resolution (Loro et al. 2016). Light-driven increases in stromal pH, driven by translocation of H^+^ into the thylakoid lumen, also have the potential to affect many fluorescent proteins. Earlier reports suggested stromal pH alkalizes strongly in the light (from pH 7 to pH 8), although more recent studies using pH-sensitive fluorescent dyes in isolated chloroplasts from pea or *Arabidopsis* indicate a more modest increase from pH 7.3 in the dark to pH 7.6 in the light (Su and Lai 2017; Aranda Sicilia et al. 2021). pH sensitivity is an important consideration for single wavelength intensiometric calcium reporters, such as R-GECO1, which have shown much promise for highly sensitive measurements of [Ca^2+^]_cyt_ elevations in plants (Keinath et al. 2015). However, substantial oscillations of cytosolic pH in pollen tubes, guard cells, or mesophyll cells had no visible effects on cytosolic R-GECO1 (Li et al. 2021). Measurements of cytosolic Ca^2+^ during pH oscillations in *Arabidopsis* pollen tubes showed that jR-GECO1a gave similar results to the pH-insensitive Ca^2+^ sensor yellow cameleon YC3.6 (Guo et al. 2022). R-GECO1-based Ca^2+^ indicators have previously been used to measure Ca^2+^ dynamics in organelles, such as the mitochondria of animal cells, although they exhibited some sensitivity to large increases in pH induced by 30 mm NH_4_Cl (Hou et al. 2017; Kanemaru et al. 2020). YC3.6 is less sensitive to changes in pH because the individual CFP and YFP components of YC3.6 exhibit a similar degree of pH sensitivity, resulting in little change in the CFP/YFP ratio (Keinath et al. 2015). Direct imaging of stromal-localized YC3.6 has been used to successfully demonstrate [Ca^2+^]_str_ elevations during light to dark transitions in *Arabidopsis* leaves, and revealed complex additional dynamics such as fast Ca^2+^ spikes in single chloroplasts (Loro et al. 2016).

A recent study using imaging of YC3.6 in the green alga *Chlamydomonas reinhardtii* indicated that [Ca^2+^]_str_ elevations could be induced by exposure to high light (Pivato et al. 2023). The amplitude and timing of the [Ca^2+^]_str_ elevations were dependent on light intensity and were not affected by the removal of external Ca^2+^, suggesting that they originated from an intracellular Ca^2+^ source. Pivato et al. (2023) demonstrated that light intensities used to induce [Ca^2+^]_str_ elevations caused an accumulation of H_2_O_2_ in the chloroplast (measured using the fluorescent H_2_O_2_ sensor roGFP2-Tsa2ΔC_R_) and that [Ca^2+^]_str_ elevations could be directly induced by 1 mm H_2_O_2_ in the absence of a light stimulus. These findings suggest that [Ca^2+^]_str_ elevations play a role in the response of green algae to oxidative stress induced by high light.

Ca^2+^ signaling within chloroplasts has not yet been explored in diatoms. Photosynthetic stramenopiles acquired their plastids via an endosymbiotic association with a red alga and their plastids demonstrate important organizational differences from those found in the Archaeplastida. Diatom plastids are surrounded by 4 membranes, with the outer membrane connected to the endoplasmic reticulum (ER) (Falciatore et al. 2020). The thylakoids are not organized into grana, but instead form loose stacks of 3 vesicles containing the fucoxanthin–chlorophyll–protein light-harvesting complexes (Flori et al. 2017). Photoprotection in diatoms utilizes a highly efficient fast-responding nonphotochemical quenching (NPQ) component that is dependent on a modified xanthophyll cycle involving the interconversion of diadinoxanthin (Ddx) and diatoxanthin (Dtx) (Lavaud and Kroth 2006). These structural and functional differences suggest that many signaling processes associated with diatom chloroplasts are likely to be unique.

*Phaeodactylum tricornutum* is a genetically amenable pennate diatom that represents the model species for many aspects of diatom biology, although *P. tricornutum* lacks the characteristic silicified frustule found in most other diatoms (Falciatore et al. 2020). *P. tricornutum* was originally isolated from rock pools on the coast of the UK and has subsequently been identified in a broad range of coastal and brackish locations (De Martino et al. 2007). It is, therefore, likely to experience substantial variability in physical parameters such as light, temperature, and salinity within its natural habitat. Initial studies using aequorin demonstrated the presence of [Ca^2+^]_cyt_ elevations in response to hypo-osmotic stress, mechanical stimulation, and the addition of iron and the diatom aldehyde decadienal (Falciatore et al. 2000; Vardi et al. 2006). More recently, expression of R-GECO1 in *P. tricornutum* and the centric diatom *Thalassiosira pseudonana* has enabled the visualization of [Ca^2+^]_cyt_ elevations in single diatom cells in response to membrane depolarization, hypo-osmotic stress, cold temperature, and the supply of phosphate (Helliwell et al. 2019, 2021a, 2021b; Kleiner et al. 2022). Diatoms, therefore, exhibit robust cytosolic calcium signaling responses to environmental stimuli, but the involvement of the chloroplast in these responses remains unknown.

In this study, we have examined the nature of chloroplast Ca^2+^ signaling in diatoms. We expressed the intensiometric reporters G-GECO1 and R-GECO1 and the ratiometric reporter G-GECO1-mApple in the chloroplast stroma of *P. tricornutum.* Our results show that [Ca^2+^]_str_ acts independently of [Ca^2+^]_cyt_ and that both high light and oxidative stress induce large sustained [Ca^2+^]_str_ elevations in diatoms. By expressing fluorescent reporters for H_2_O_2_, we demonstrate that [Ca^2+^]_str_ elevations coincide with the accumulation of H_2_O_2_ within the chloroplast.

## Results

### Treatments that induce cytosolic ca^2+^ elevations in *P. tricornutum* do not induce stromal Ca^2+^ elevations

**Figure 1.**
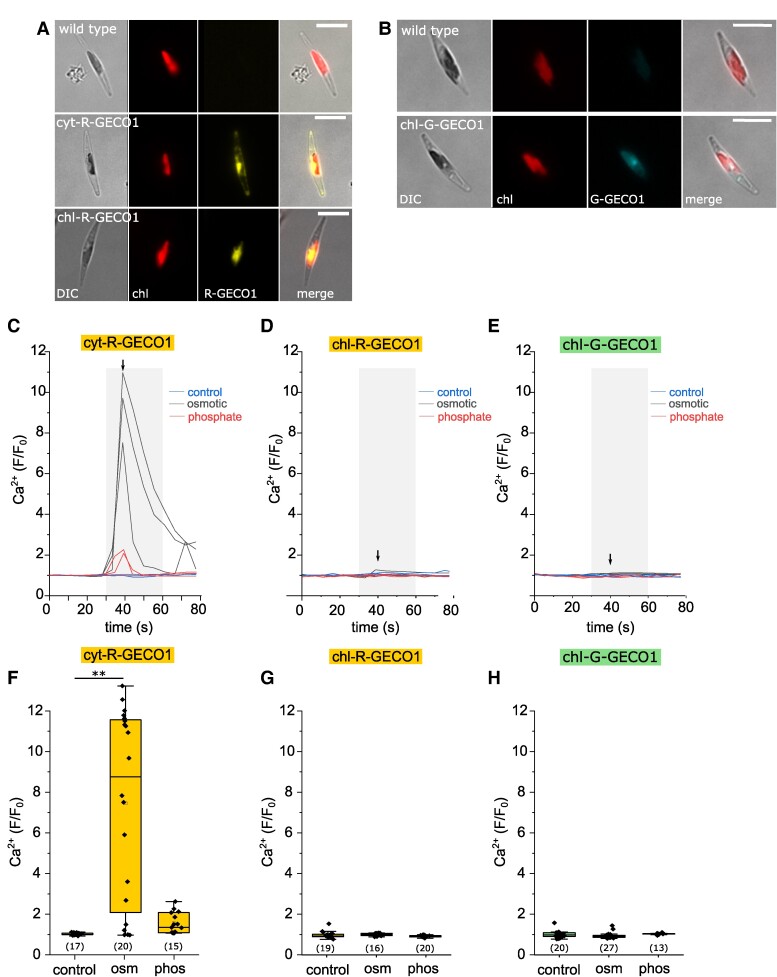
Cytosolic Ca^2+^ elevations occur independently from stromal Ca^2+^. **A)** Epifluorescent microscopy images of cells expressing R-GECO1 in the cytosol (cyt-R-GECO1) or chloroplast stroma (chl-R-GECO1). DIC = differential interference contrast, chl = chlorophyll autofluorescence. Bar =10 µm. **B)** Cells expressing G-GECO1 in the chloroplast stroma (chl-G-GECO1). Bar = 10 µm. **C)** Changes in cytosolic Ca^2+^ (cyt-R-GECO1) following hypo-osmotic shock (dilute artificial seawater, 75% ASW) or the addition of inorganic phosphate to P-limited cells (36 µM added to cells grown at 1.8 µM Pi for 4 d). Three representative traces are shown for each treatment. The shaded area indicates the duration of treatment (30–60 s), arrow indicates the time point used for the box plots **(F–H)**. Each treatment was repeated at least three times on three independent cell cultures. Ct = control, Osm = hypo-osmotic shock, phos = resupply of inorganic phosphate. **D)** No changes were observed in stromal Ca^2+^ (chl-R-GECO1) following hypo-osmotic shock or the addition of inorganic phosphate. Three representative traces are shown for each treatment. **E)** As in **(D)** but with chl-G-GECO1 cells. Three representative traces are shown for each treatment. **F)** Box plots show the amplitude of [Ca^2+^]_cyt_ elevations at *t* = 40 s (10 s after treatment) as change in fluorescence (*F*/*F*_0_). The box plot indicates interquartile range (IQR) (25–75%), whiskers 1.5 IQR. The median (line) and mean (open square) are also shown. The number of cells is listed in parentheses. ** = mean value is significantly different from the control (*P* < 0.01, one-way ANOVA, Tukey post hoc). **G)** Amplitude for [Ca^2+^]_str_ increases after 40 s (10 s treatment) measured using chl-R-GECO1. The box plot indicates interquartile range (IQR) (25–75%), whiskers 1.5 IQR. The median (line) and mean (open square) are also shown. **H)** Amplitude for [Ca^2+^]_str_ increases after 40 s (10 s treatment), measured using chl-G-GECO1. The box plot indicates interquartile range (IQR) (25–75%), whiskers 1.5 IQR. The median (line) and mean (open square) are also shown.

We generated strains expressing calcium indicators targeted to the chloroplast stroma through fusion of R-GECO1 or G-GECO1 to the plastid-targeting presequence of oxygen-evolving enhancer protein (Oee1) (Gruber et al. 2007) (Fig. 1, A and B). The reporters exhibited a clear chloroplast localization and were not influenced by chlorophyll autofluorescence (Supplementary Fig. S1). We did not obtain a strain expressing cytosol-localized G-GECO1. In land plants, many stimuli that induce [Ca^2+^]_cyt_ elevations, such as oxidative stress and NaCl, also induce rapid [Ca^2+^]_str_ elevations (Sello et al. 2018). We, therefore, compared *P. tricornutum* cells expressing R-GECO1 in the cytosol or chloroplast following treatments that induce robust [Ca^2+^]_cyt_ elevations (Helliwell et al. 2021a, 2021b). Application of hypo-osmotic shock (75% artificial seawater, ASW) resulted in large [Ca^2+^]_cyt_ elevations in 75% of cells (mean *F*/*F*_0_ 7.48 ± 4.71), but did not lead to any [Ca^2+^]_str_ elevations (Fig. 1, C to H). Addition of 36 µM PO_4_^3−^ to phosphate-limited cells caused smaller transient [Ca^2+^]_cyt_ elevations in 47% of cells (mean *F*/*F*_0_ 1.55 ± 0.52), but did not cause [Ca^2+^]_str_ elevations (Fig. 1, C to H). Observations using the alternative Ca^2+^ reporter strain chl-G-GECO1 confirmed the absence of [Ca^2+^]_str_ elevations in response to these stimuli (Fig. 1, E and H). Our findings indicate that [Ca^2+^]_str_ in *P. tricornutum* is not directly influenced by large [Ca^2+^]_cyt_ transients, suggesting that [Ca^2+^]_str_ can act independently from [Ca^2+^]_cyt_.

### Light stress induces specific [Ca^2+^]_str_ elevations that are not observed in the cytosol

**Figure 2.**
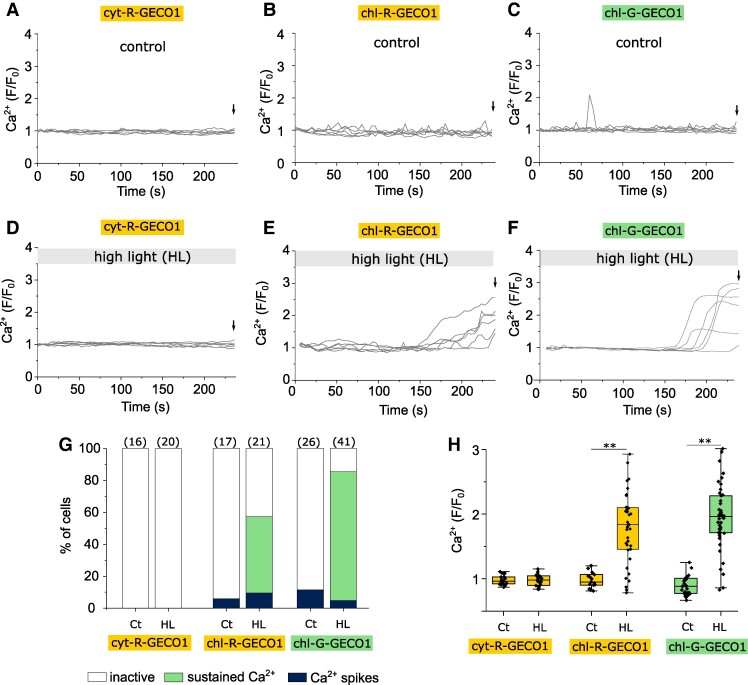
High light causes an increase in chloroplast Ca^2+^. Effect of excitation light on cells expressing cytosolic (cyt-R-GECO1) or chloroplast (chl-R-GECO1 or chl-G-GECO1) localized calcium reporters. Six representative cells are shown for each treatment. **A)** Cytosolic Ca^2+^ (*F*/*F*_0_) in cyt-R-GECO1 cells under control imaging conditions (intermittent excitation every 4 s, mean irradiance 451 μmol m^−2^ s^−1^). The arrow indicates the time point used for the box plots (4 min, shown in **(H)**). Six representative cells are shown (*n* = 16). **B)** chl-R-GECO1 cells under control imaging conditions. Six representative cells are shown (*n* = 17). **C)** chl-G-GECO1 cells under control imaging conditions. Note that occasional spontaneous [Ca^2+^]_str_ elevations are observed. Six representative cells are shown (*n* = 26). **D)** Imaging of [Ca^2+^]_cyt_ under high light conditions (intermittent excitation with higher intensity 470 nm LED, mean irradiance 2365 μmol m^−2^ s^−1^ from time 0 s). No [Ca^2+^]_cyt_ elevations were observed. Six representative cells are shown (*n* = 20). **E)** Imaging of chl-R-GECO1 cells under high light conditions resulted in large sustained [Ca^2+^]_str_ elevations. The missing values at the start of the timecourse were due to focus adjustment. Six representative cells are shown (*n* = 21). **F)** chl-G-GECO1 cells also show a sustained increase in [Ca^2+^]_str_ under high light conditions. Six representative cells are shown (*n* = 41). **G)** Percentage of cells classed as inactive (no Ca^2+^ elevation), showing Ca^2+^ spikes (Ca^2+^ elevations <10 s), or sustained Ca^2+^ elevations (>10 s) at control imaging conditions (Ct) and high light (HL). The number of cells examined is shown in parentheses. **H)** Ca^2+^ elevations after imaging for 4 min. The change in fluorescence (*F*/*F*_0_) for all 3 reporters after 4 min is shown, number of cells as in **(G)**. ** = mean values are significantly different from control (*P* < 0.01, one-way ANOVA, Tukey post hoc). The box plot indicates interquartile range (IQR) (25–75%), whiskers 1.5 IQR. The median (line) and mean (open square) are also shown.

Previous studies using fluorescent indicators in plants have used intermittent excitation (e.g. 5 s intervals) at minimal intensity to prevent activation of photosynthesis (measured through the formation of the trans-thylakoid pH gradient) (Loro et al. 2016; Su and Lai 2017; Ugalde et al. 2021). Our control imaging conditions (intermittent excitation every 4 s), revealed no [Ca^2+^]_cyt_ elevations within a 4 min period, but identified occasional [Ca^2+^]_str_ elevations in a small proportion of cells (Fig. 2, A to C, Supplementary Fig. S2). Imaging at higher irradiances (intermittent excitation every 4 s at a higher intensity, mean irradiance 2,365 μmol m^−2^ s^−1^) had a substantial impact on [Ca^2+^]_str_ (Fig. 2, D to F). 62% of chl-R-GECO1 cells exhibited a large sustained (>10 s) increase in [Ca^2+^]_str_ within the 4 min observation period, whereas no changes were observed in [Ca^2+^]_cyt_ in cells expressing cyt-R-GECO1 (Fig. 2, G and H). 80.5% of cells expressing chl-G-GECO1 also showed a sustained [Ca^2+^]_str_ elevation within this time period. [Ca^2+^]_str_ elevations were not inhibited by the removal of external Ca^2+^ (Ca^2+^-free ASW + 200 μM EGTA) (Supplementary Fig. S3). Removing external Ca^2+^ inhibits [Ca^2+^]_cyt_ elevations in response to osmotic, cold, and phosphate treatments in *P. tricornutum* (Helliwell et al. 2019, 2021a, 2021b). The [Ca^2+^]_str_ elevations, therefore, do not require entry of external Ca^2+^ across the plasma membrane and must utilize internal Ca^2+^ stores. As [Ca^2+^]_cyt_ in eukaryotes is maintained at very low concentrations and does not become elevated during high light stress in *P. tricornutum*, the most likely source of the [Ca^2+^]_str_ elevations is release of Ca^2+^ from the thylakoid lumen or chloroplast ER.

To confirm that the large [Ca^2+^]_str_ elevations induced by excess excitation light do not influence [Ca^2+^]_cyt_, we generated a dual reporter strain expressing distinct Ca^2+^ reporters in each compartment (R-GECO1 in the cytosol and G-GECO1 in the chloroplast) (Supplementary Fig. S4). 85% of cells expressing the dual reporters exhibited a [Ca^2+^]_str_ elevation in response to high light but no cells exhibited a [Ca^2+^]_cyt_ elevation.

### [Ca^2+^]_str_ elevations exhibit distinct spatial and temporal properties

**Figure 3.**
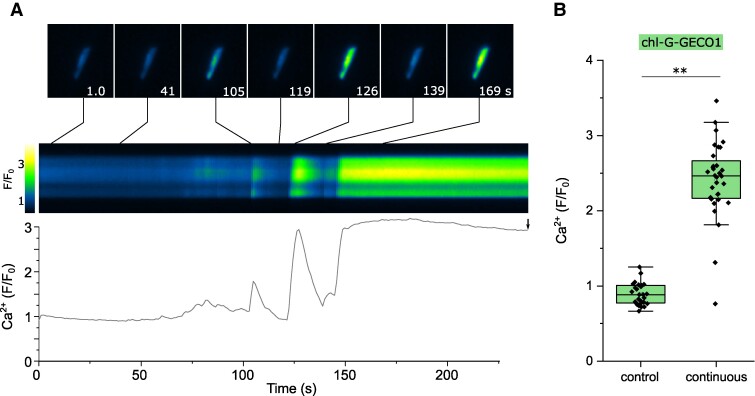
Continuous illumination with blue light results in dynamic [ca^2+^]_str_ elevations. **A)** chl-G-GECO1 fluorescence observed under continuous excitation (470 nm at standard intensity, mean irradiance 1,860 μmol m^−2^ s^−1^). In the example shown, several transient [Ca^2+^]_str_ elevations can be observed prior to a sustained [Ca^2+^]_str_ elevation around 150 s. The trace indicates fold change in fluorescence over time, with the kymograph indicating the change in fluorescence along a longitudinal transect through the chloroplast over this period. Selected individual images from the time course are shown (top panel). Bar = 5 µm. The arrow indicates the time point used for the box plots (4 min, shown in **B)**. **B)** [Ca^2+^]_str_ (change in chl-G-GECO1 fluorescence) is shown after 4 min for cells imaged under control conditions (intermittent excitation) and continuous illumination. ** = significantly different from control (*P* < 0.01, one-way ANOVA, Tukey post hoc). *n* = 27 and 32 cells, respectively. The box plot indicates interquartile range (IQR) (25–75%), whiskers 1.5 IQR. The median (line) and mean (open square) are also shown.

We next examined whether continuous illumination of chl-G-GECO1 cells at standard intensity (470 nm mean irradiance 1,860 μmol m^−2^ s^−1^) also induced [Ca^2+^]_str_ elevations. This approach allowed us to examine [Ca^2+^]_str_ elevations at higher temporal resolution. Continuous illumination induced a sustained increase in [Ca^2+^]_str_ in 100% of chl-G-GECO1 cells within 4 min (Fig. 3). Closer inspection of these traces revealed complex dynamics of [Ca^2+^]_str_, with multiple rapid Ca^2+^ transients preceding the eventual sustained [Ca^2+^]_str_ elevation. Moreover, [Ca^2+^]_str_ elevations exhibited spatial properties, with distinct differences in the timing of [Ca^2+^]_str_ elevations within different regions of the chloroplast (Supplementary Fig. S5). This suggests that stimuli can be perceived locally and the information propagated to the rest of the chloroplast through a [Ca^2+^]_str_ elevation. The thylakoids in diatom chloroplasts are primarily distributed around the periphery of the plastid with a central pyrenoid acting as the primary site for CO_2_ fixation. Localized [Ca^2+^]_str_ elevations could therefore be associated with suborganellar structures, such as the pyrenoid, as has been proposed in *C. reinhardtii* (Wang et al. 2016).

We conclude that excess excitation light, either through intermittent illumination at high intensity or continuous illumination at a lower intensity, leads to a sustained elevation of [Ca^2+^]_str_. Rapid Ca^2+^ spiking preceding a sustained [Ca^2+^]_str_ elevation was previously observed in individual *Arabidopsis* chloroplasts, although this occurred following a light to dark transition (Loro et al. 2016).

### Chloroplast Ca^2+^ elevations can be induced by oxidants

**Figure 4.**
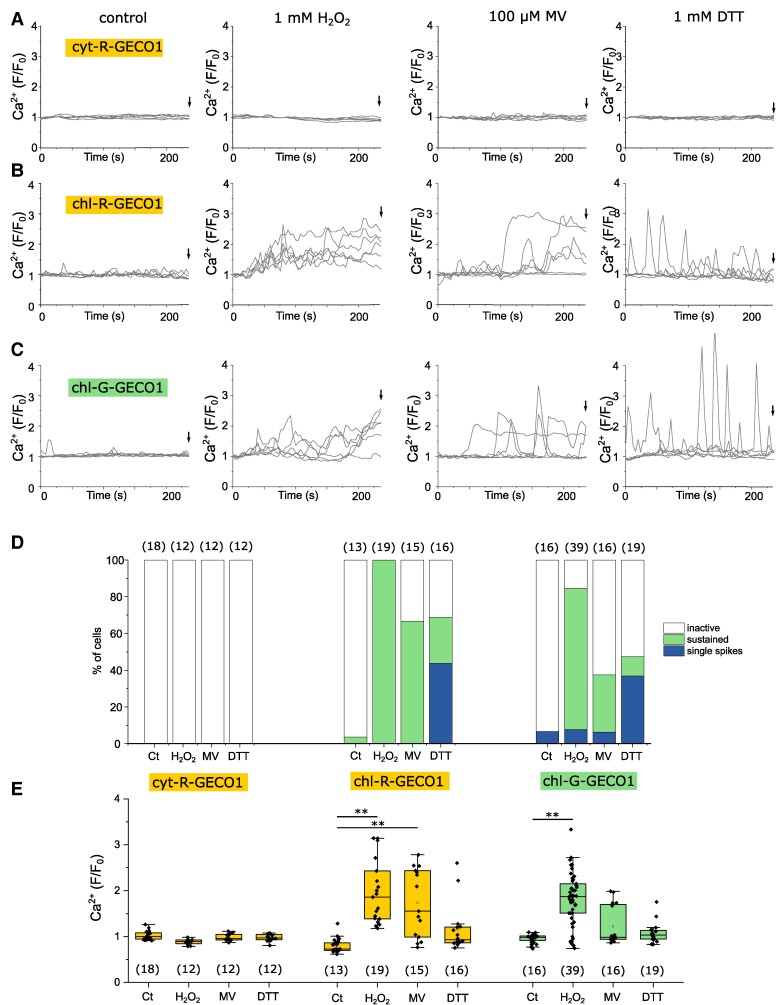
Oxidative stress induces sustained [Ca^2+^]_str_ elevations. **A)** [Ca^2+^]_cyt_ measured in cyt-R-GECO1 cells exposed to the oxidants H_2_O_2_ and methyl viologen (MV) and the reducing agent dithiothreitol (DTT). Treatments applied at time 0 s. Six representative traces are shown for each treatment (total 18, 12, 12, 12 cells, respectively, shown in **E)**. None of the treatments lead to [Ca^2+^]_cyt_ elevations. The arrow indicates the time point used for the box plots (4 min, shown in **E)**. **B)** [Ca^2+^]_str_ measured with chl-R-GECO1 cells in response to the treatments described in **(A)**. All treatments result in significant [Ca^2+^]_str_ elevations, although 1 mm DTT leads to transient Ca^2+^ spikes rather than sustained [Ca^2+^]_str_ elevations. The arrow indicates the time point used for the box plots (4 min, shown in **E)**. Six representative traces are shown for each treatment (total 13, 19, 15, 16 cells, respectively, shown in **E)**. **C)** [Ca^2+^]_str_ measured with chl-G-GECO1 cells shows a very similar response to H_2_O_2_, MV and DTT. The arrow indicates the time point used for the box plots (4 min, shown in **E)**. Six representative traces are shown for each treatment (total 16, 39, 16, 19 cells, respectively, shown in **E)**. **D)** Percentage of cells classed as inactive (no Ca^2+^ elevation), showing Ca^2+^ spikes (Ca^2+^ elevations with a duration <10 s), or sustained Ca^2+^ elevation (duration >10 s) for each treatment. The number of cells examined is shown in parentheses. **E)** Change in fluorescence from initial (*F*/*F*_0_) for each treatment after 4 min. Number of cells as in **(D)**. ** = mean amplitude at 4 min significantly different from control, *P* < 0.01 (one-way ANOVA, Tukey post hoc).

In photosynthetic organisms, illumination with high light can overwhelm cellular antioxidant defences and lead to an accumulation of reactive oxygen species (ROS) in the chloroplast. We hypothesized that the accumulation of ROS may contribute to the [Ca^2+^]_str_ elevations induced by high light in *P. tricornutum*. Application of 1 mm hydrogen peroxide (H_2_O_2_) triggered substantial [Ca^2+^]_str_ elevations in chl-R-GECO1 cells that were similar in amplitude to those induced by high light, but did not induce [Ca^2+^]_cyt_ elevations (Fig. 4, A and B).

Methyl viologen (MV) competes with ferredoxin to accept electrons from photosystem I resulting in the formation of superoxide in the chloroplast, which is subsequently converted to H_2_O_2_ by action of superoxide dismutase (Kozuleva et al. 2021). In *Arabidopsis*, MV results in a rapid accumulation of H_2_O_2_ in the chloroplast, with slower and less pronounced elevations in H_2_O_2_ observed in other cellular compartments (Ugalde et al. 2021). These researchers found that intermittent illumination during fluorescent imaging was sufficient to cause the generation of ROS in MV-treated plants. *P. tricornutum* treated with 100 μM MV exhibited substantial [Ca^2+^]_str_ elevations in 66.7% of chl-R-GECO1 cells (imaged under standard conditions with no additional illumination) but did not exhibit [Ca^2+^]_cyt_ elevations (Fig. 4, A and B). [Ca^2+^]_str_ elevations can, therefore, be induced by a treatment that generates ROS primarily within the chloroplast.

We next tested whether addition of a reducing agent (dithiothreitol, DTT), which prevents accumulation of ROS, inhibited [Ca^2+^]_str_ elevations. Cells treated with 1 mm DTT for 4 min demonstrated a series of transient [Ca^2+^]_str_ elevations although they did not exhibit a sustained increase in [Ca^2+^]_str_. The nature of the Ca^2+^ signaling response to DTT was distinct from those induced by high light, H_2_O_2_, and MV (Fig. 4, A and B). The results suggest that [Ca^2+^]_str_ elevations can be induced by perturbation of chloroplast redox state, either to a more oxidized or a more reduced state, although the nature of the elevations is distinct. In addition to sequestering ROS, the addition of DTT will influence many other redox-sensitive processes. DTT is commonly used in diatoms to disrupt NPQ through inhibition of the redox-sensitive xanthophyll cycle enzyme violaxanthin de-epoxidase (VDE), which catalyzes the de-epoxidation of diadinoxanthin (Blommaert et al. 2021).

We also identified [Ca^2+^]_str_ elevations in response to H_2_O_2_, MV and DTT in chl-G-GECO1 cells, indicating that the responses can be detected via an alternative Ca^2+^ reporter (Fig. 4, C to E).

### Ratiometric Ca^2+^ sensors for chloroplast signaling

Single wavelength Ca^2+^ indicators are subject to a series of limitations, such as artifacts caused by cell movement. As the [Ca^2+^]_str_ elevations in *P. tricornutum* are associated with excess light, changes in stromal pH could also influence R-GECO1 and G-GECO1 fluorescence (emission intensity increases as pH rises) (Zhao et al. 2011). The magnitude of the fluorescence changes in response to Ca^2+^ are likely to be far greater than the pH effect (Li et al. 2021) and we have applied a stringent threshold (*F*/*F*_0_ > 1.5 fold) when identifying [Ca^2+^]_str_ elevations. However, it is important that studies of organellar Ca^2+^ dynamics address these potential issues.

The ratiometric Ca^2+^ sensor YC3.6 used previously to monitor [Ca^2+^]_str_ in plants is largely insensitive to pH because the 2 fluorophores exhibit a similar pH sensitivity (Loro et al. 2016). Unfortunately, we were unable to successfully target YC3.6 to *P. tricornutum* chloroplasts. We, therefore, fused G-GECO1 to mApple, which is insensitive to Ca^2+^ but shows mild sensitivity to pH (Shen et al. 2014; Rennick et al. 2022), to allow ratiometric imaging of Ca^2+^ with reduced pH sensitivity. Cytosolic G-GECO1-mApple showed robust responses to hypo-osmotic stress (Supplementary Fig. S6). When exposed to strong perturbation of cytosolic pH through the addition of 10 mm NH_4_Cl, the ratiometric sensor exhibits a small degree of sensitivity to pH, although its sensitivity to Ca^2+^ is far greater.

**Figure 5.**
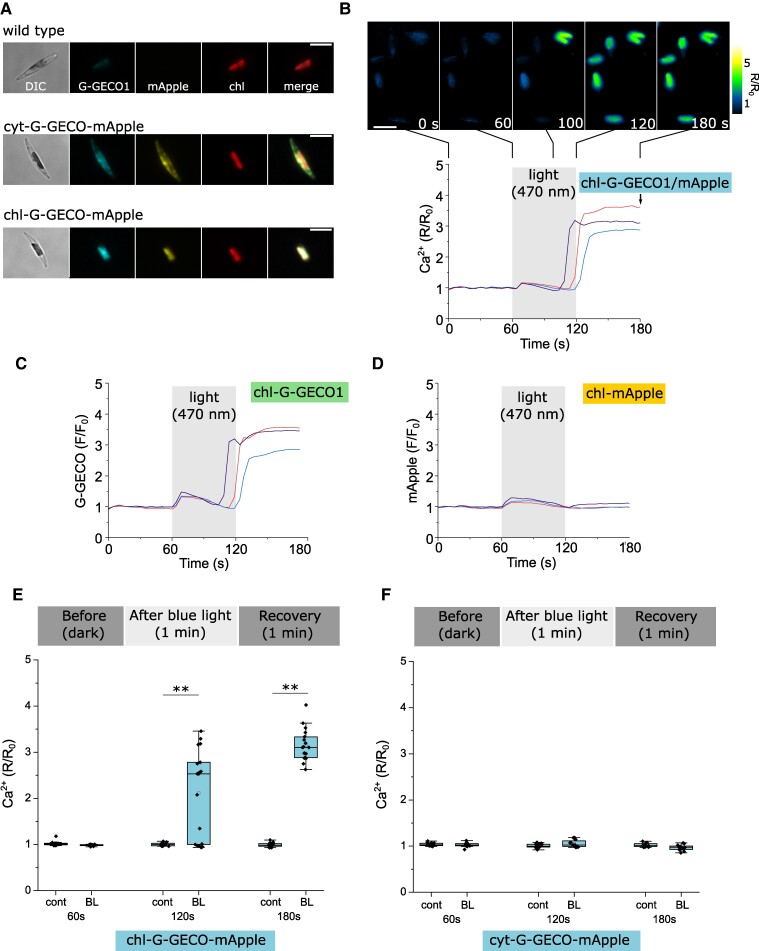
The use of a ratiometric Ca^2+^ indicator to image [Ca^2+^]_str_ elevations caused by continuous illumination. **A)** Epifluorescent microscopy images showing cells expressing the ratiometric Ca^2+^ indicator G-GECO1-mApple in the cytosol or chloroplast stroma. Chl = chlorophyll autofluorescence, DIC = differential interference contrast. Bar = 5 µm. **B)** [Ca^2+^]_str_ elevations caused by continuous illumination. Cells were imaged under control conditions (intermittent excitation every 5 s) for 60 s and then exposed to continuous blue light illumination for a further 60 s (mean irradiance 4194 μmol m^−2^ s^−1^). A rapid rise in the ratio of chl-G-GECO1-mApple fluorescence (*R*/*R*_0_) occured at the end of the period of continuous illumination or during the return to control imaging conditions. Three representative traces are shown, with false color images of chl-G-GECO1-mApple cells shown above. Bar = 5 µm. The arrow indicates the time point used for the box plots (3 min, shown in **E)**. **C)** Changes in G-GECO1 fluorescence from individual cells shown in **(B)**. **D)** Changes in mApple fluorescence from individual cells shown in **(B)**. The small increase in mApple fluorescence during the period of continuous light is likely due to an increase in stromal pH, whilst the large sustained increases in G-GECO1 fluorescence are absent from mApple. **E)** Changes in [Ca^2+^]_str_ after continuous light (*t =* 120 s) and dark recovery period (*t =* 180 s). ** = mean values are significantly different from control, *P* < 0.01 (one-way ANOVA, Tukey post hoc). *n* = 15 (control) and 18 (continuous light) cells. **F)** Changes in [Ca^2+^]_cyt_ measured in cyt-G-GECO1-mApple cells following continuous illumination. *n* = 18 (control) and 12 (continuous light) cells. No [Ca^2+^]_cyt_ elevations were observed.

We expressed the ratiometric Ca^2+^ sensor in the chloroplast and examined its response to continuous light. We imaged chl-G-GECO1-mApple cells under standard conditions for 60 s (intermittent excitation), before switching to continuous illumination for 60 s (470 nm, mean irradiance 4,194 μmol m^−2^ s^−1^), followed by 60 s of recovery. The irradiance used was the lowest we could achieve for blue excitation light with the imaging system (1% LED power with 10% transmission through neutral density filters). 60 s of continuous illumination caused large sustained [Ca^2+^]_str_ elevations in all cells (*n* = 17) (Fig. 5, A to D). [Ca^2+^]_str_ did not elevate immediately on illumination but rose rapidly toward the end of the illumination period or even in the dark recovery period. 61% of cells exhibited sustained [Ca^2+^]_str_ elevations after 1 min of illumination, rising to 100% of cells following 1 further minute of dark recovery (Fig. 5E). Imaging of the cells over a longer period indicated that [Ca^2+^]_str_ remained elevated for >10 min in all cells before returning to resting values in 62% of cells after 25 min (*n* = 21) (Supplementary Fig. S7). Illumination of cyt-G-GECO1-mApple cells with continuous light for 60 s did not cause [Ca^2+^]_cyt_ elevations (Fig. 5F).

To explore the effect of pH on the chloroplast-localized reporter, we perfused chl-G-GECO1-mApple cells with 10 mm NH_4_Cl. NH_4_Cl is commonly used as a tool to manipulate cytosolic pH, as NH_3_ readily crosses the plasma membrane and forms NH_4_^+^ in the cytosol, consuming a H^+^ (Boron 2004). In photosynthetic tissues, NH_4_Cl has an important additional impact, acting to uncouple the photosynthetic electron transport chain from ATP generation, due to alkalization of the thylakoid lumen and disruption of the trans-thylakoid H^+^ gradient (Dean and Miskiewicz 2003). Acidification of the thylakoid lumen is important for the activation of NPQ in diatoms, and treatment of *P. tricornutum* with 5 mm NH_4_Cl severely inhibits NPQ (Blommaert et al. 2021) (Supplementary Fig. S8). Treatment of the chl-G-GECO1-mApple strain with 10 mm NH_4_Cl for >60 s led to large sustained [Ca^2+^]_str_ elevations in 75% of cells. The [Ca^2+^]_str_ elevations could result from a direct influence of stromal alkalinization on Ca^2+^ transport processes, e.g. by modifying the activity of Ca^2+^/H^+^ exchangers, but may also result from disruption of the trans-thylakoid H^+^ gradient by NH_4_Cl and the subsequent inhibition of NPQ. Alkalinization of the cytosol by NH_4_Cl did not cause elevations in [Ca^2+^]_cyt_ (Supplementary Fig. S6) indicating that this response is specific to the chloroplast.

**Figure 6.**
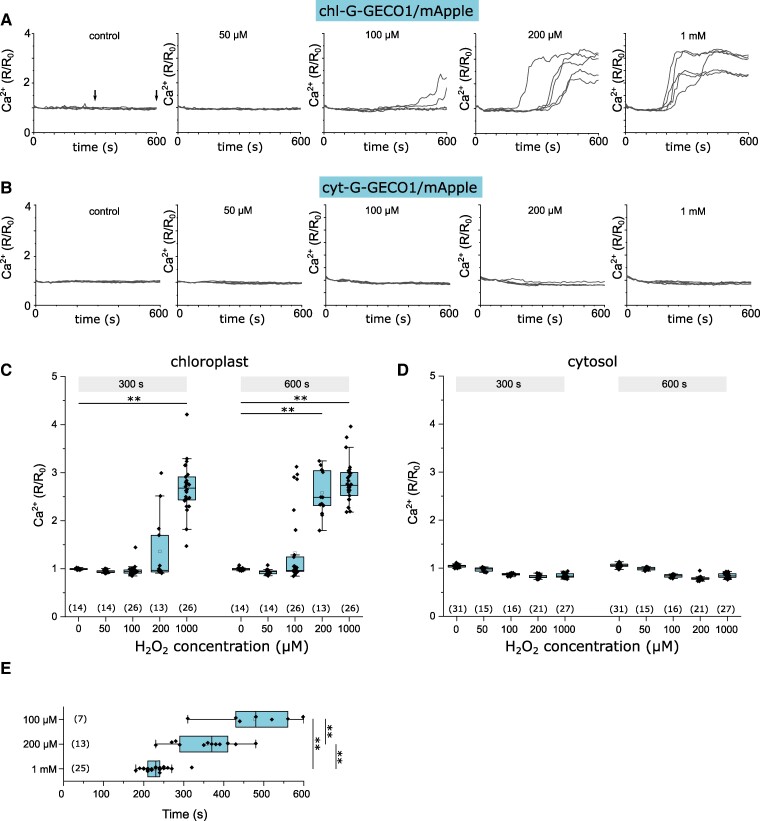
[Ca^2+^]_str_ elevations induced by exogenous H_2_O_2_. **A)** Representative traces from 5 individual cells indicating changes in chl-G-GECO1-mApple fluorescence ratio in response to exogenous H_2_O_2_ added at time 0 s. H_2_O_2_ caused a sustained increase in [Ca^2+^]_str_ at higher concentrations. The arrows indicate the time points used for the box plots (shown in **C**). **B)** Changes in cyt-G-GECO1-mApple fluorescence ratio in response to exogenous H_2_O_2_. No changes in [Ca^2+^]_cyt_ were observed. Representative traces from 5 individual cells are shown. **C)** Box plots showing chl-G-GECO1-mApple fluorescence ratio in response to exogenous H_2_O_2_ after 300 and 600 s. ** = mean values are significantly different from control, *P* < 0.01 (one-way ANOVA, Tukey post hoc). The box plot indicates interquartile range (IQR) (25–75%), whiskers 1.5 IQR. The median (line) and mean (open square) are also shown. **D)** Changes in cyt-G-GECO1-mApple fluorescence ratio in response to exogenous H_2_O_2_ after 300 and 600 s. The box plot indicates interquartile range (IQR) (25–75%), whiskers 1.5 IQR. The median (line) and mean (open square) are also shown. **E)** Timing of [Ca^2+^]_str_ elevations (*R*/*R*_0_ > 1.5) in response to exogenous H_2_O_2._ ** = mean values are significantly different from other treatments, *P* < 0.01 (one-way ANOVA, Tukey post hoc). The box plot indicates interquartile range (IQR) (25–75%), whiskers 1.5 IQR. The median (line) and mean (open square) are also shown.

We next used the ratiometric Ca^2+^ indicator to explore the response of [Ca^2+^]_str_ and [Ca^2+^]_cyt_ to a wider range of H_2_O_2_ concentrations. Application of exogenous H_2_O_2_ to chl-G-GECO-mApple cells resulted in sustained [Ca^2+^]_str_ elevations in 100% of cells treated with 200 μM and 1 mm H_2_O_2_ (Fig. 6, A to D). Only 27% of cells responded to 100 μM H_2_O_2_ and no [Ca^2+^]_str_ elevations were observed at 50 μM H_2_O_2_. [Ca^2+^]_str_ elevations occurred more rapidly at the higher H_2_O_2_ concentrations (Fig. 6E). No [Ca^2+^]_cyt_ elevations were observed following the addition of H_2_O_2_ to cyt-G-GECO-mApple cells (Fig. 6B). The absence of a response to H_2_O_2_ in the cytosol with either G-GECO-mApple or R-GECO also suggests that H_2_O_2_ does not have a direct impact on the Ca^2+^ reporters themselves.

### [Ca^2+^]_str_ elevations correspond to increased H_2_O_2_ concentrations within the chloroplast

**Figure 7.**
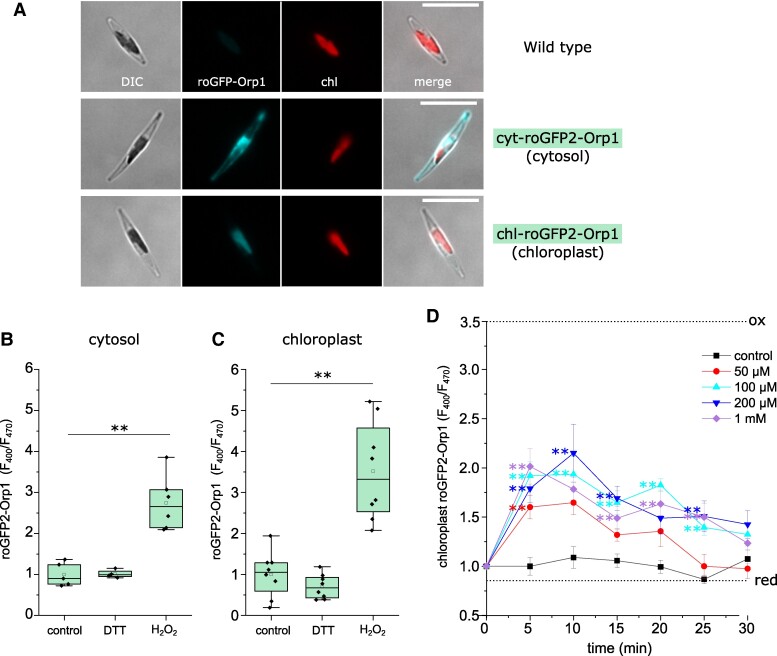
Measurement of H_2_O_2_ in the cytosol and chloroplast using roGFP2-Orp1. **A)** Epifluorescent microscopy images of *P. tricornutum* cells expressing the fluorescent H_2_O_2_ reporter roGFP2-Orp1 in the cytosol (middle panel) and chloroplast (lower panel), compared to wild type cells (upper panel). DIC = differential interference contrast, chl = chlorophyll autofluorescence. Bar = 10 µm. **B)** Determination of cytosolic roGFP2-Orp1 fluorescence ratio (*F*_400_/*F*_470_) when fully oxidized (1 mm H_2_O_2_) or fully reduced (1 mm dithiothreitol, DTT). Measurements were taken using a fluorescent plate reader after treatment had been applied for 5 min. ** = mean value is significantly different from control, *P* < 0.01 (one-way ANOVA, Tukey post hoc). *n* = 8 replicates. **C)** Determination of chloroplast roGFP2-Orp1 fluorescence ratio after treatment with 1 mm H_2_O_2_ or 1 mm DTT. *n* = 8 replicates. **D)** Time course of chloroplast H_2_O_2_ after addition of exogenous H_2_O_2_. Chloroplast roGFP2-Orp1 fluorescence ratio was measured at 5 min intervals using a plate reader assay. ** indicates mean values are significantly different from control at each time point, *P* < 0.01 (one-way ANOVA, Tukey post hoc). *n* = 8 replicates, error bars represent Se. Dotted lines indicate mean *F*_400_/*F*_470_ for fully oxidized and fully reduced probes.

Fluorescent reporters can be used to examine the redox status of different cellular compartments, allowing a closer examination of the relationship between chloroplast H_2_O_2_ and [Ca^2+^]_str_ elevations. Using a chloroplast targeted roGFP, which primarily reports the oxidation status of the glutathione pool (*E*_GSH_), van Creveld et al. (2015) demonstrated that 80 μM of exogenous H_2_O_2_ oxidized *P. tricornutum* chloroplasts after 30 min. To measure H_2_O_2_ directly, we expressed the ratiometric fluorescent biosensor roGFP2-Orp1 in the cytosol or chloroplast of *P. tricornutum* (Fig. 7A). roGFP2-Orp1 is largely insensitive to changes in pH and is highly specific for H_2_O_2_ over other ROS (although it can also be oxidized by peroxynitrite) (Nietzel et al. 2019). Cytosolic- and chloroplast-localized roGFP2-Orp1 reporters were oxidized by 1 mm H_2_O_2_, but were not significantly reduced by the addition of 1 mm DTT, indicating that the resting levels of H_2_O_2_ are low within both of these compartments (Fig. 7, B and C).

Addition of a wide range of H_2_O_2_ concentrations (50 μM to 1 mm) led to significant oxidation of chloroplast-localized roGFP2-Orp1 in all treatments within 5 min (Fig. 7D). chl-roGFP2-Orp1 remained oxidized in the 100 μM, 200 μM, and 1 mm H_2_O_2_ treatments after 15 min, but no treatments were significantly different from the control after 30 min. Thus, all treatments cause an increase in chloroplast H_2_O_2_, but the magnitude and duration of the increase depends upon the concentration of exogenous H_2_O_2_. As only the higher concentrations of exogenous H_2_O_2_ cause [Ca^2+^]_str_ elevations, these observations suggest that [Ca^2+^]_str_ elevations are triggered by the accumulation of chloroplast H_2_O_2_ beyond a threshold value.

### Exogenous H_2_O_2_ has transient effects on photophysiology, but leads to defects in growth

**Figure 8.**
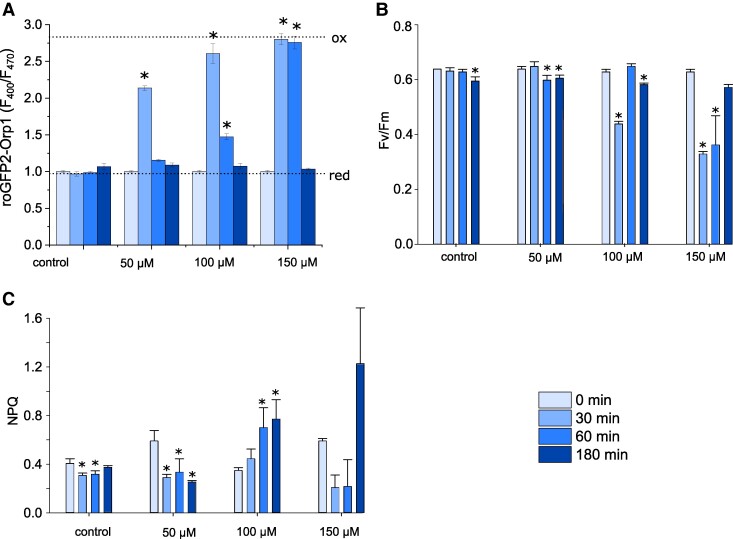
Effect of exogenous H_2_O_2_ on photophysiology. **A)** Cytosolic H_2_O_2_ levels measured in a plate reader assay using roGFP2-Orp1 after addition of different concentrations of H_2_O_2_ over a 3 h period. *n* = 3 biological replicates, mean values, error bars represent Sd. * = significantly different from the initial value for each treatment (*P* < 0.05, one-way ANOVA, Tukey post hoc). These statistical tests were applied to B and C also. Dotted lines indicate *F*_400_/*F*_470_ for fully oxidized (ox) and fully reduced (red) probes. **B)** Photosynthetic efficiency of photosystem II (*F*_v_/*F*_m_) in the same cells described in **(A)**. *n* = 3 biological replicates, mean values, error bars represent Sd. **C)** Measurement of nonphotochemical quenching (NPQ) in the same cells described in **(A)**. *n* = 3 biological replicates, mean values, error bars represent Sd.

We examined how long-term exposure to these H_2_O_2_ concentrations influenced photosynthesis and growth in *P. tricornutum*. Mizrachi et al. (2019) demonstrated that concentrations of H_2_O_2_ greater than 80 μM caused long-lasting oxidation of the chloroplast within a sub-population of *P. tricornutum* cells that led to their death within 24 h. We exposed *P. tricornutum* to 3 concentrations of H_2_O_2_ (50, 100, and 150 μM) and photosynthetic parameters were measured over 3 h using pulse-amplitude-modulated (PAM) fluorimetry. These experiments were performed in cells expressing cyt-roGFP2-Orp1, so that we could directly determine the duration and amplitude of cellular H_2_O_2_ stress at each concentration. Cytosolic H_2_O_2_ was strongly elevated in all treatments 30 min after the addition of exogenous H_2_O_2_, although it returned to resting values after 3 h, with the intensity and duration of the elevation correlating to the concentration of H_2_O_2_ applied (Fig. 8A). The photosynthetic efficiency of photosystem II (*F*_v_/*F*_m_) did not change following the addition of 50 µM H_2_O_2_, although there was a significant, but transient, decline in *F*_v_/*F*_m_ after treatment with 100 and 150 µM H_2_O_2_ (Fig. 8B). Therefore, both cytosolic H_2_O_2_ and *F*_v_/*F*_m_ were restored to resting values after 3 h treatment, suggesting that low concentrations of exogenous H_2_O_2_ may only have transient impacts on photosynthesis. However, NPQ was significantly elevated after 3 h at 100 μM H_2_O_2_, indicating that the exposure to oxidative stress had a longer lasting impact on photophysiology, even after cytosolic H_2_O_2_ had returned to resting values (Fig. 8C). Moreover, growth was severely impaired at all treatments above 50 µM in a dose-dependent manner, indicating that whilst damage to the photosystem was temporary, the oxidative stress experienced was sufficient to inhibit cell division and/or cause cell death (Supplementary Fig. S9). Therefore, it seems that the initial high levels of H_2_O_2_ cause physiological changes that persist after the added H_2_O_2_ has been detoxified by antioxidant defences.

### Light-induced [Ca^2+^]_str_ elevations are linked to the accumulation of chloroplast H_2_O_2_

**Figure 9.**
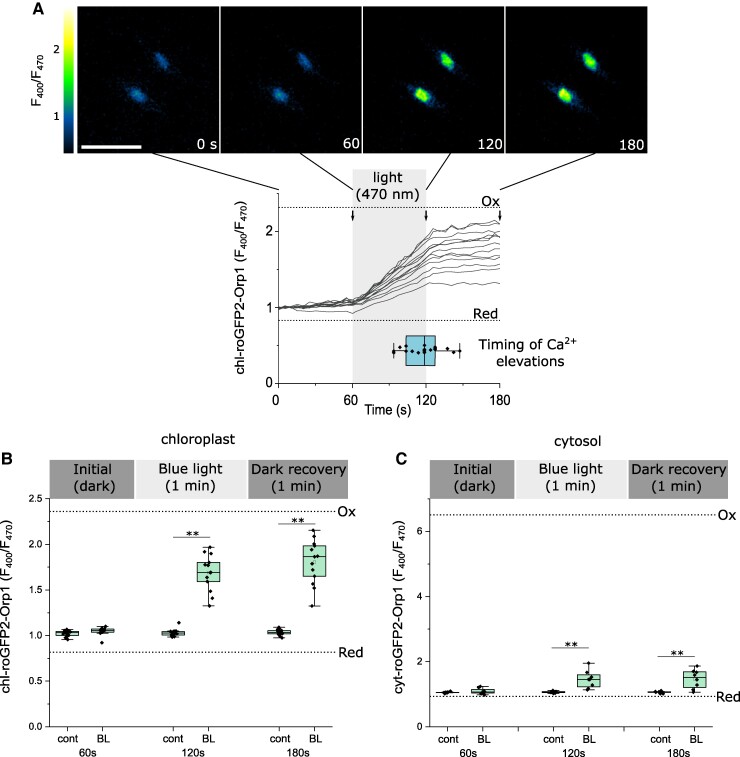
Continuous illumination leads to an increase in chloroplast H_2_O_2_. **A)** Time course microscopy of individual chl-roGFP2-Orp1 cells exposed to 60 s of continuous light at. Cells were imaged under control conditions for 60 s and then imaged with continuous blue light (470 nm) from 60 to 120 s (conditions that caused [Ca^2+^]_str_ elevations in Fig. 5). Representative traces from chl-roGFP2-Orp1 cells showing a gradual accumulation of H_2_O_2_ in each cell during high light stress (*n* = 13 cells). Dotted lines indicate *F*_400_/*F*_470_ for fully oxidized (1 mm H_2_O_2_) and fully reduced probes (1 mm dithiothreitol, DTT). Dotted lines indicate *F*_400_/*F*_470_ for fully oxidized (ox) and fully reduced (red) probes. The superimposed horizontal box plot indicates the timing of [Ca^2+^]_str_ elevations from chl-G-GECO1-mApple cells exposed to the same treatment (Fig. 5). The box plot indicates interquartile range (IQR) (25–75%), whiskers 1.5 IQR. The median (line) and mean (open square) are also shown. The arrows indicate the time points used for the box plots (shown in **(B)**). **B)** Box plots showing changes in chloroplast H_2_O_2_ in individual cells following exposure to continuous light. *n* = 19 (control) and 13 (continuous light). ** = mean value is significantly different from the control, *P* < 0.01 (one-way ANOVA, Tukey post hoc). These statistical tests were applied to C also. The box plot indicates interquartile range (IQR) (25–75%), whiskers 1.5 IQR. The median (line) and mean (open square) are also shown. **C)** Changes in cytosolic H_2_O_2_ in individual cells following exposure to continuous light. *n* = 9 (control) and 8 (continuous light). The box plot indicates interquartile range (IQR) (25–75%), whiskers 1.5 IQR. The median (line) and mean (open square) are also shown.

We next examined whether high irradiances that induce [Ca^2+^]_str_ elevations also result in an accumulation of H_2_O_2_ in the chloroplast by imaging chl-roGFP2-Orp1 cells under identical conditions to those used to induce [Ca^2+^]_str_ elevations in chl-G-GECO1-mApple cells (continuous blue light, 470 nm at 4,194 μmol m^−2^ s^−1^ for 60 s) (Fig. 5). Chloroplast H_2_O_2_ remained stable under standard imaging conditions (intermittent excitation every 5 s) but increased steadily in all cells during the period of continuous illumination (Fig. 9, A and B, Supplementary Fig. S10). We also observed a small but significant increase in cytosolic H_2_O_2_ after the blue light stress (Fig. 9C). chl-roGFP2-Orp1 remained oxidized following the return to intermittent excitation. The return of oxidized roGFP probes to resting values is determined by the rate at which they are reduced by thiol-based systems in each compartment. In plant cells, the redox potential of the glutathione pool (*E*_GSH_) is the primary determinant of the rate of reduction of oxidized roGFP2-Orp1 (Nietzel et al. 2019). The gradual increase in chloroplast H_2_O_2_ from the onset of illumination contrasts with the delayed timing of the [Ca^2+^]_str_ elevations (mean 58.0 ± 15.7 s after the initiation of light stress, *n* = 18) (Fig. 4). The steady increase in H_2_O_2_ in the chloroplast during light stress suggests that accumulation of H_2_O_2_ above a threshold value is necessary to initiate [Ca^2+^]_str_ elevations.

**Figure 10.**
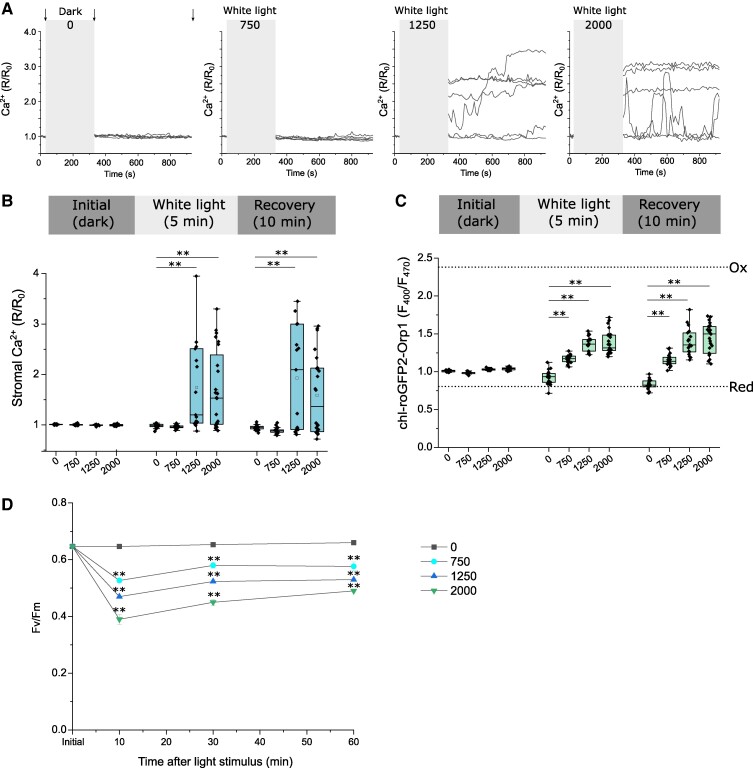
Effect of high light on Ca^2+^ and H_2_O_2_ in the chloroplast and photosynthetic efficiency. **A)** Changes in [Ca^2+^]_str_ following exposure to continuous white light (0; 750; 1,250; 2,000 μmol m^−2^ s^−1^). chl-G-GECO-mApple cells were imaged for 30 s and then exposed to white light for 5 min (grey box, no Ca^2+^ imaging was possible during this period). The cells were then imaged for a further 10 min to follow changes in [Ca^2+^]_str_. Six representative traces are shown (*n* = 20, 17, 15, and 24, respectively). Arrows indicate timings chosen for box plots (shown in **B)**. **B)** Box plots summarizing the experiments shown in **(A)**. The change in [Ca^2+^]_str_ is shown before treatment, immediately after exposure to light (5 min) and then after 10 min of dark recovery. ** = significantly different from control (dark treated) value, *P* < 0.01 (one-way ANOVA, Tukey post hoc). *n* = 20, 17, 15, and 24, respectively. The box plot indicates interquartile range (IQR) (25–75%), whiskers 1.5 IQR. The median (line) and mean (open square) are also shown. **C)** Box plots showing changes in chloroplast H_2_O_2_ following exposure to 5 min of continuous white light (0; 750; 1,250; 2,000 μmol m^−2^ s^−1^). Individual chl-roGFP2-Orp1 cells were exposed to light as described in **(A)**. Fluorescence ratio was determined before treatment, immediately after exposure to light (5 min) and then after 10 min dark recovery. ** = significantly different from control (dark treated) value, *P* < 0.01 (one-way ANOVA, Tukey post hoc). *n* = 17, 20, 19, and 21, respectively. The box plot indicates interquartile range (IQR) (25–75%), whiskers 1.5 IQR. The median (line) and mean (open square) are also shown. Dotted lines indicate *F*_400_/*F*_470_ for fully oxidized (ox) and fully reduced (red) probes. **D)** Mean maximum quantum efficiency of PSII (*F*_v_/*F*_m_) following exposure to 5 min of continuous white light (0; 750; 1,250; 2,000 μmol m^−2^ s^−1^). Time represents period of dark recovery. ** = mean values are significantly different from the control value (dark), *P* < 0.01 (one-way ANOVA, Tukey post hoc). Error bars represent Sd, *n* = 3.

The use of excitation light (470 nm) to induce light stress allowed direct imaging of Ca^2+^ and H_2_O_2_ dynamics during the period of illumination. However, excitation light is highly focused by the microscope objective, resulting in a high irradiance that may exceed those encountered under natural conditions. To examine cellular responses to a more environmentally relevant light stress, we exposed *P. tricornutum* cells to a wide range of irradiances using a diffuse white light source. We found that exposure to 750 μmol m^−2^ s^−1^ for 5 min did not induce [Ca^2+^]_str_ elevations, but higher irradiances (1,250 and 2,000 μmol m^−2^ s^−1^) led to substantial [Ca^2+^]_str_ elevations in 53% and 58% of cells, respectively (Fig. 10, A and B). In many cells [Ca^2+^]_str_ became elevated during the light stress and remained elevated during 10 min of dark recovery, although some cells exhibited dynamic changes in [Ca^2+^]_str_ during the recovery period. Application of the same irradiances to chl-roGFP2-Orp1 cells indicated a large accumulation of chloroplast H_2_O_2_ at the higher irradiances (1,250 and 2,000 μmol m^−2^ s^−1^) and a small but significant increase in chloroplast H_2_O_2_ at the lower irradiance (750 μmol m^−2^ s^−1^) (Fig. 10C). As the lower irradiance did not cause [Ca^2+^]_str_ elevations, this further supports the hypothesis that accumulation of H_2_O_2_ above a threshold concentration may be needed to trigger [Ca^2+^]_str_ elevations. All 3 light treatments caused a significant decrease in photosynthetic performance, measured as maximum quantum yield of PSII (*F*_v_/*F*_m_) (Fig. 10D). However, all treatments exhibited a partial recovery of *F*_v_/*F*_m_ within 1 h of the application of light stress, indicating that these irradiances were unlikely to result in irreversible damage to the photosynthetic apparatus. The light treatments did not cause [Ca^2+^]_cyt_ elevations, further indicating that [Ca^2+^]_str_ acts independently of [Ca^2+^]_cyt_ (Supplementary Fig. S11). We observed a significant increase in cytosolic H_2_O_2_ at the highest irradiance (2,000 μmol m^−2^ s^−1^), but not at 750 or 1,250 μmol m^−2^ s^−1^ (Supplementary Fig. S11). Therefore, light stress causes a specific increase in chloroplast H_2_O_2_ at lower irradiances. It seems likely that the increase in cytosolic H_2_O_2_ at the highest irradiance is caused by excess H_2_O_2_ diffusing from the chloroplast.

Treatment of *P. tricornutum* with 10 µM 3-(3,4-dichlorophenyl)-1,1-dimethylurea (DCMU) to block photosynthetic electron transport between photosystem II and plastoquinone did not inhibit [Ca^2+^]_str_ elevations caused by 5 min of white light (2,000 μmol m^−2^ s^−1^) (Supplementary Fig. S12). However, the interpretation of this result is complex, because while DCMU blocks direct generation of H_2_O_2_ (Exposito-Rodriguez et al. 2017) it can also lead to the formation of highly reactive singlet oxygen in both plants and diatoms (Flors et al. 2006). Therefore, treatment with DCMU does not prevent photo-oxidative stress but represents an alternative source of ROS. Indeed, we found that oxidation of chl-roGFP2-Orp1 following exposure to high light was greater after treatment with 10 µM DCMU (Supplementary Fig. S12). A similar result was observed in *C. reinhardtii* using chloroplast-localized roGFP2-Tsa2ΔCR (Pivato et al. 2023). A recent study in demonstrated that although DCMU blocks direct H_2_O_2_ production in land plants it can lead to the formation of lipid peroxides, which are also detected by roGFP2-Orp1 (Exposito-Rodriguez et al. 2024).

## Discussion

Our findings indicate that diatom plastids possess Ca^2+^ signaling mechanisms that exhibit many similarities to those found in green algae. Both *P. tricornutum* and *C. reinhardtii* exhibit [Ca^2+^]_str_ elevations in response to high light with the amplitude dependent on the irradiance used (Fig. 10) (Pivato et al. 2023). However, there are a number of distinctions between [Ca^2+^]_str_ elevations in diatom chloroplasts and those observed in the primary plastids of land plants. Multiple stimuli that cause [Ca^2+^]_cyt_ elevations in land plants also cause [Ca^2+^]_str_ elevations (Sello et al. 2016, 2018). In contrast, we did not observe [Ca^2+^]_str_ elevations in *P. tricornutum* in response to stimuli that induce [Ca^2+^]_cyt_ elevations. Robust [Ca^2+^]_str_ elevations in *P. tricornutum* were observed in response to oxidants (H_2_O_2_ and MV), to the reducing agent DTT, to NH_4_Cl and to excess light. Whilst light and H_2_O_2_ also induce [Ca^2+^]_str_ elevations in land plants, there are important differences. 10 mm H_2_O_2_ induces Ca^2+^ elevations in both the chloroplast and the cytosol of *Arabidopsis* cell cultures (Sello et al. 2016), whereas *P. tricornutum* exhibits Ca^2+^ elevations exclusively in the chloroplast in response to lower concentrations of H_2_O_2_ (50 µM to 1 mm) (Fig. 7). Light-related [Ca^2+^]_str_ elevations in land plants occur following a transition from light to dark (Loro et al. 2016). In contrast, [Ca^2+^]_str_ elevations *P. tricornutum* were caused by exposure to high light intensities and can occur during the light stimulus. This points to important differences between the chloroplast Ca^2+^ signaling pathways of land plants and those found in diatoms and green algae.

[Ca^2+^]_str_ elevations in *P. tricornutum* were only observed at higher irradiances (Fig. 10), suggesting that they represent a high light response, rather than a general response to light or to light of a specific wavelength (e.g. blue light). However, we cannot discount a spectral component to the response, as high light-induced [Ca^2+^]_str_ elevations in *C. reinhardtii* were reduced in cryptochrome mutants (Pivato et al. 2023). Our evidence suggests that the [Ca^2+^]_str_ elevations induced by high light in *P. tricornutum* are linked to the formation of ROS in the chloroplast. Irradiances that elicited [Ca^2+^]_str_ elevations also led to the accumulation of H_2_O_2_ in the chloroplast and [Ca^2+^]_str_ elevations could also be induced directly by oxidants. However, milder treatments that cause significant elevations in chloroplast H_2_O_2_, such as lower irradiances (750 μmol m^−2^ s^−1^ white light) or 50 μM H_2_O_2_ do not trigger [Ca^2+^]_str_ elevations. Moreover, only a sub-population of cells exhibit [Ca^2+^]_str_ elevations at higher irradiances, although chloroplast H_2_O_2_ becomes elevated in all cells. These findings suggest that accumulation of chloroplast H_2_O_2_ above a threshold concentration is required to trigger [Ca^2+^]_str_ elevations. *P. tricornutum* is found in intertidal and coastal locations in temperate regions, where surface irradiance can often exceed 1,000 μmol m^−2^ s^−1^ for prolonged periods in summer months (Kolzenburg et al. 2023). As these irradiances can lead to oxidation of the chloroplast redox state in *P. tricornutum* (Mizrachi et al. 2019), diatoms are likely to regularly experience conditions that induce photo-oxidative stress within their natural environment. Estuarine and littoral diatoms, such as *P. tricornutum*, exhibit a much greater tolerance of high light than coastal or oceanic species (Lavaud et al. 2007)*. P. tricornutum* is able to rapidly fine-tune its photosynthetic and photoprotective mechanisms to tolerate episodes of high light (up to 2,000 μmol m^−2^ s^−1^) (Lavaud et al. 2007; Eisenstadt et al. 2008; Domingues et al. 2012). Its ability to rapidly induce high capacity mechanisms of dissipating excess light energy suggests *P. tricornutum* is primarily adapted to low light environments that experience periodic exposure to very high irradiances (Lavaud et al. 2007).

The [Ca^2+^]_str_ elevations induced by NH_4_Cl (Supplementary Fig. S8) may also be due to the accumulation of ROS, as this treatment leads to inhibition of NPQ (Blommaert et al. 2021). NPQ is a major mechanism through which excess excitation energy is dissipated, so inhibition of NPQ can also result in the accumulation of ROS, even at low light intensities (Roach et al. 2015). Disruption of qE (the pH-dependent component of NPQ) in the *npq4* mutant of *Chlamydomonas* leads to a substantial elevation in total cellular H_2_O_2_ compared to wild type cells (Allorent et al. 2013). 1 mm DTT also inhibits NPQ, but this treatment prevents any increase in chloroplast H_2_O_2_. As DTT induced Ca^2+^ spiking rather than sustained [Ca^2+^]_str_ elevations, transient [Ca^2+^]_str_ elevations may result from the inhibition of a redox-sensitive process (such as NPQ), whereas sustained [Ca^2+^]_str_ elevations may result from the accumulation of ROS. These complex signaling events clearly require further dissection, but it seems likely that [Ca^2+^]_str_ elevations reflect information assimilated from multiple inputs in order to provide a coordinated downstream response.

Chloroplast Ca^2+^ signaling has been implicated in the regulation of photosynthesis via several mechanisms. In the green alga *C. reinhardtii,* chloroplast Ca^2+^ signaling modulates photosynthetic electron transport by regulating cyclic electron flow (CEF) around photosystem I, contributing to both the PGRL1/PGR5 and the NAD(P)H dehydrogenase (NDH)-dependent CEF pathways (Hochmal et al. 2015). These responses are mediated by the Ca^2+^ sensor protein CAS, which localizes to the thylakoid membrane and forms a complex with Proton Gradient Regulation Like 1 (PGRL1) and Anaerobic Response 1 (ANR1) (Terashima et al. 2012). CAS also regulates the expression of LHCSR3, a light-harvesting protein that is required for the dissipation of excess light energy through NPQ (Petroutsos et al. 2011). Ca^2+^ therefore regulates the activity of both CEF and NPQ in *C. reinhardtii.* As CAS also regulates the activity of the carbon concentrating mechanism in *C. reinhardtii* (Wang et al. 2016), chloroplast Ca^2+^ signaling plays a central role in coordinating cellular responses to light and inorganic carbon. CAS also plays a role in photoacclimation and stomatal closure in land plants, although its role is less clear. CAS abundance increases in high light in plants and is a target for phosphorylation by the state transition kinase 8 (STN8), which influences CEF (Li et al. 2022). However, CAS is absent from diatom genomes, so the mechanisms of chloroplast Ca^2+^ signaling must differ substantially in this lineage.

The link between [Ca^2+^]_str_ elevations and photo-oxidative stress suggests that chloroplast Ca^2+^ signaling could play a role in the photoprotective response of diatoms. As NPQ is induced in *P. tricornutum* by modest increases in irradiance (e.g. 18 to 135 μmol m^−2^ s^−1^) (Blommaert et al. 2021) that do not cause [Ca^2+^]_str_ elevations, Ca^2+^ is most likely involved in photoprotective responses to more severe light stress. Several lines of evidence support a role for chloroplast Ca^2+^ signaling in diatom photoprotection. NPQ requires establishment of the transthylakoidal proton gradient, which activates the VDE enzyme to promote the formation of diatoxanthin (Dtx) (Lavaud and Kroth 2006). In plants, the transthylakoidal H^+^ gradient is modulated by the activity of a conserved family of K^+^/H^+^ antiporters, KEA1-3 (Kunz et al. 2014; Wang et al. 2017), and a KEA3 homolog also acts as a major regulator of NPQ in *P. tricornutum* (Seydoux et al. 2022). Interestingly, KEA3 from *P. tricornutum* and other diatoms differs from plant KEA3 proteins in that they possess a pair of Ca^2+^-binding EF-hands at the C-terminus. Mutant *P. tricornutum* lines expressing a truncated KEA3 without EF-hands exhibit a similar NPQ phenotype to *kea3* knock-out mutants, indicating that the ability to bind Ca^2+^ is essential for its role in photoprotection (Seydoux et al. 2022). *Arabidopsis* KEA3 is orientated so that it mediates H^+^ efflux from the thylakoid lumen (i.e. it lowers the H^+^ gradient when active) with the C-terminal region situated in the stroma (Wang et al. 2017). Assuming PtKEA3 orientates in a similar manner, its EF-hands would be positioned to detect [Ca^2+^]_str_ elevations, such as those caused by high light and oxidative stress.

Several other Ca^2+^-binding proteins have been identified in diatom chloroplasts (Liu et al. 2022). These include DSP1 (death-specific protein), a protein that was first identified as being upregulated during cell death in *Skeletonema costatum* (Chung et al. 2005). DSP proteins localize to the chloroplast and show weak similarity to the plant thylakoid-associated proton gradient regulator-5 (PGR5), which contributes to CEF around photosystem I (Thamatrakoln et al. 2013). All diatom DSP proteins contain a C-terminal pair of EF-hands, suggesting that their activity is also regulated by changes in [Ca^2+^]_str_ (Chung et al. 2008). DSP proteins appear to play an important role in the responses of diatoms to iron limitation, with *T. pseudonana* lines overexpressing TpDSP1 showing increased growth under iron limitation due to elevated CEF (Thamatrakoln et al. 2013; Hao et al. 2021). The presence of Ca^2+^-binding domains in the key photoprotective proteins KEA3 and DSP1, in combination with the pronounced [Ca^2+^]_str_ elevations in response to high light, support an important role for chloroplast Ca^2+^ signaling in diatom photoprotection.

Ca^2+^ signaling also plays an important role in programmed cell death (PCD) in diatoms (Vardi et al. 2006). The individual components that mediate PCD are not yet known, although diatoms possess several metacaspases, a family of Ca^2+^-dependent proteases associated with PCD in other eukaryotes. Recent characterization of a type III metacaspase from *P. tricornutum* (PtMCA-IIIc) revealed that it is co-regulated by Ca^2+^ and redox status (Graff van Creveld et al. 2021). Given that exogenous H_2_O_2_ at concentrations of 80–200 µM can oxidize the chloroplast and lead to cell death in *P. tricornutum* (Mizrachi et al. 2019), it is possible that [Ca^2+^]_str_ elevations induced by oxidants are also linked to the PCD signaling pathway. Both the prolonged chloroplast oxidation (Mizrachi et al. 2019) and the [Ca^2+^]_str_ elevations (this study) exhibit a threshold-like response that was restricted to a sub-population of cells at lower concentrations of H_2_O_2_. However, direct evidence linking [Ca^2+^]_str_ elevations to PCD signaling is not yet available, as the localization of PtMCA-IIIc remains unknown and PCD signaling caused by the diatom-derived aldehyde decadienal was linked to [Ca^2+^]_cyt_ elevations, rather than chloroplast signaling (Vardi et al. 2006).

Diatom chloroplasts, like those of land plants, can act as autonomous Ca^2+^ signaling organelles. [Ca^2+^]_str_ elevations most likely result from release of Ca^2+^ from the thylakoid lumen or the chloroplast ER (the outermost membrane of the chloroplast that is continuous with the outer nuclear envelope and the ER). Significant recent progress in plants has identified several chloroplast-localized Ca^2+^ transporters. *Arabidopsis* BICAT1 and BICAT2 are related to the yeast Ca^2+^ transporter Gdt1 and localize to the thylakoid membrane or the chloroplast envelope, respectively (Frank et al. 2019). Disruption of the BICAT proteins leads to substantial changes in the [Ca^2+^]_str_ transients invoked by light to dark shifts (Frank et al. 2019). In addition, a member of the mitochondrial calcium uniporter family has been shown to localize to the chloroplast in *Arabidopsis* (cMCU), where it contributes to [Ca^2+^]_str_ dynamics in response to osmotic stress (Teardo et al. 2019). Plastid-localized glutamate receptors GLR3.4 and GLR3.5 also contribute to Ca^2+^ entry from the cytosol (Teardo et al. 2011, 2015). The entire MCU complex is missing from diatoms (Pittis et al. 2020) and GLRs are also absent from *P. tricornutum* (Verret et al. 2010), although BICAT proteins are present. The mechanisms mediating Ca^2+^ entry into diatom plastids may therefore differ substantially from those in land plants.

Our results reveal a highly dynamic Ca^2+^ signaling system within diatom chloroplasts that can act independently of cytosolic Ca^2+^ signaling pathways. [Ca^2+^]_str_ elevations can be induced by a variety of stimuli linked to photo-oxidative stress, suggesting that chloroplast Ca^2+^ signaling may play an important role in diatom photoprotection. Important distinctions in the nature of chloroplast Ca^2+^ signaling between diatoms and land plants, and in the mechanisms through which Ca^2+^ signals are generated and sensed, suggest that diatoms possess unique signaling mechanisms to regulate photosynthetic function.

## Materials and methods

### Strains and culturing conditions

The wild type *P. tricornutum* strain used in this study was CCAP 1055/1 (Culture Collection of Algae and Protozoa, SAMS, Oban, UK). Cultures were maintained in natural seawater with f/2 nutrients (Guillard 1975). For imaging experiments, cells were acclimated to an ASW medium for minimum 10 days prior to analysis. ASW contained 450 mm NaCl, 30 mm MgCl_2_, 16 mm MgSO_4_, 8 mm KCl, 10 mm CaCl_2_, 2 mm NaHCO_3_, 97 µM H_3_BO_3_, f/2 supplements and 20 mm HEPES (pH 8.0). Cultures were grown at 18 °C with a 16:8 light/dark cycle under an irradiance of 50 µmol m^−2^ s^−1^ and used in mid-exponential phase.

### Generation of *P. tricornutum* strains expressing fluorescent Ca^2+^ and H_2_O_2_ reporters

*P. tricornutum* transformed with the cytosolic R-GECO1 Ca^2+^ biosensor (PtR1) was described previously (Helliwell et al. 2019). All other strains were generated in this study. G-GECO1 and R-GECO1 are intensiometric Ca^2+^ reporters that emit green or yellow/orange fluorescence, respectively (Zhao et al. 2011). roGFP2-Orp1 is a ratiometric redox sensor that is highly selective for H_2_O_2_ over other forms of ROS (Gutscher et al. 2009). To create plasmids for expression of G-GECO1, R-GECO1 and roGFP2-Orp1 in the cytosol, codon optimized genes were synthesized (Genscript, Netherlands, GenBank Accession OR136874-7) and cloned into the pPha-T1 expression vector via *EcoRI* and *BamHI* restriction sites. Additional constructs for chloroplast-localized biosensors were created by inserting the chloroplast-targeting sequence from *OEE1* into the *EcoRI* site of these plasmids. Oee1 is the oxygen-evolving enhancer that stabilizes the oxygen-evolving complex in the thylakoid membrane. Its presequence has been used previously to target fluorescent proteins to the chloroplast stroma (Gruber et al. 2007; Rosenwasser et al. 2014). The G-GECO1-mApple fusion, with the addition of a GGGSGGGS glycine linker between the 2 proteins and the *OEE1* chloroplast-targeting sequence, was synthesized (Genscript) and cloned into the pPha-T1 expression vector via *EcoRI* and *BamHI* restriction sites. Biolistic transformation of *P. tricornutum* was performed as described in (Helliwell et al. 2019).

### Epifluorescence imaging of biosensors

500 µL of cell culture was added to a 35 mm microscope dish with glass coverslip base (In Vitro Scientific, Sunnyvale, CA, USA) coated with 0.01% poly-L-lysine (Merck Life Science UK, Gillingham, Dorset) to promote cell adhesion to the glass surface. Cells were allowed to settle for 5–20 min at room temperature (21 °C). R-GECO1 and G-GECO1 were imaged using a Leica DMi8 inverted microscope (Leica Microsystems, Milton Keynes, UK) with a 63× 1.4NA oil immersion objective. A SpectraX LED light source (Lumencor) was used with a 550/15 nm (center wavelength/bandwidth) excitation filter (4% LED intensity) and 585/40 nm emission filter for R-GECO1 and a 470/24 nm excitation filter (4% LED intensity) and 525/50 nm emission filter for G-GECO1. Images were captured with a Prime 95B sCMOS camera (Teledyne Photometrics, Birmingham, UK) (4 s intervals, 900 ms exposure) using LasX software v.3.3.0 (Leica). Imaging of G-GECO1-mApple used the Leica DMi8 setup but with a PE-300ultra LED light source (CoolLED). Sequential excitation was applied (470/24 nm, 200 ms, 1% LED intensity and 550/15 nm, 200 ms, 2% LED intensity) every 5 s. For continuous light experiments, the excitation shutter was left open after capturing each frame, resulting in continuous illumination by the 470 nm excitation light, although the camera exposure remained the same (200 ms). Imaging of the H_2_O_2_ biosensor roGFP2-Orp1 was performed using sequential ratiometric excitation at 400 nm (390/22) and 470 (470/24) nm, with a 525/50 nm emission filter. Oxidants and other chemical stimuli were administered to cells on the microscope by perfusion using a gravity-fed microfluidics setup. Stock solutions of H_2_O_2_, NH_4_Cl, DTT, and MV were prepared in deionized water, before being added to the ASW at the appropriate concentration. DCMU was prepared in ethanol as 10 mm stock solution. The intensity of the excitation light was measured using an S170C Microscope Slide Power Meter (Thor Labs). An in vivo calibration of cyt-G-GECO1-mApple was performed using 50 μM ionomycin in ASW containing 10 mm Ca^2+^, followed by perfusion with Ca^2+^-free ASW containg 50 μM ionomycin and 500 μM EGTA to determine R_max_ and R_min_, respectively. Using an in vitro K_d_ for G-GECO1 of 0.749 μM (Zhao et al. 2011), we estimate that an *R*/*R*_0_ of 2.0 would be equivalent to a [Ca^2+^]_cyt_ concentration of 0.98 μM.

### Processing of imaging data

Images were processed using LasX software (Leica). The mean fluorescence intensity (*F*) within a region of interest (ROI) encompassing each cell or chloroplast was measured over time. Background fluorescence was subtracted from all cellular F values. The change in the fluorescence intensity of G-GECO1 and R-GECO1 was then calculated by normalizing each frame to the initial value (*F*/*F*_0_). Ca^2+^ elevations were defined as any increase in *F*/*F*_0_ above a threshold value (>1.5), with sustained Ca^2+^ elevations defined as events where *F*/*F*_0_ was greater than 1.5 for >10 s. For G-GECO1-mApple the fluorescence ratio was determined after background subtraction (*F*_GG/mA_). For roGFP2-Orp1 the fluorescence ratio was determined following excitation at 400 and 470 nm (*F*_400_/*F*_470_). The maximum and minimum oxidation states of roGFP2-Orp1 were determined using 1 mm H_2_O_2_ and 1 mm DTT, respectively.

### Fluorescent plate reader assays to measure H_2_O_2_

Analyses were performed using a CLARIOstar Plus fluorescence plate reader (BMG LabTech, Aylesbury, UK). roGFP2-Orp1 was measured using excitation filters at 400/15 nm and 475/15 nm, with emission at 515/15 nm. Background fluorescence (seawater with no cells added) was subtracted from all samples. The maximum and minimum oxidation states of roGFP2-Orp1 were determined using 1 mm H_2_O_2_ and 1 mm DTT, respectively.

### Chlorophyll fluorimetry

Measurements of chlorophyll fluorescence were taken to assess the performance of the photosynthetic apparatus. The maximum quantum yield of photosystem II (*F*_v_/*F*_m_) and measurements of NPQ were determined using a Z985 AquaPen chlorophyll fluorimeter (Qubit Systems, Kingston, ON, Canada). Cells were dark adapted for 20 min before measurements for all experiments, except for the response to white light exposure, where a 10-min dark adaptation was used.

### Statistical analysis

Graphs and statistical analyses were performed using OriginPro (Origin Lab, Northampton, MA). Error bars represent standard deviation. Unless indicated otherwise, imaging experiments were repeated at least 3 times with independent cultures on different days to ensure reproducibility of the response. Statistical analyses of datasets with more than 2 groups were performed using an ANOVA followed by a Tukey post hoc test. Box plots indicate the interquartile range (25–75%) and whiskers show 1.5× interquartile range (IQR). The median (horizontal line) and mean (open square) are also shown.

### Accession numbers

Sequence data from this article can be found in the GenBank/EMBL data libraries under accession numbers OR136874-7.

## Supplementary Material

kiae591_Supplementary_Data

## Data Availability

Data available on request.

## Supplementary data

The following materials are available in the online version of this article.

**Supplementary Figure S1.** Relative fluorescence levels in Ca^2+^ reporter lines.

**Supplementary Figure S2.** Excitation protocols used during Ca^2+^ imaging.

**Supplementary Figure S3.** External Ca^2+^ is not required for the high light-induced [Ca^2+^]_str_ elevations.

**Supplementary Figure S4.** Simultaneous measurement of Ca^2+^ in the cytosol and chloroplast.

**Supplementary Figure S5.** Spatial specificity in [Ca^2+^]_str_ chl elevations.

**Supplementary Figure S6.** Characterization of the G-GECO1-mApple reporter.

**Supplementary Figure S7.** Sustained [Ca^2+^]_str_ elevations after continuous light stress.

**Supplementary Figure S8.** NH_4_Cl induces [Ca^2+^]_str_ elevations.

**Supplementary Figure S9.** Long-term impacts of exogenous H_2_O_2_ on growth and photophysiology.

**Supplementary Figure S10.** Calibration of roGFP2-Orp1 for single-cell microscopy.

**Supplementary Figure S11.** Effect of high light on Ca^2+^ and H_2_O_2_ in the cytosol.

**Supplementary Figure S12.** Effect of DCMU on Ca^2+^ and H_2_O_2_ in the chloroplast.

## Dive Curated Terms

The following phenotypic, genotypic, and functional terms are of significance to the work described in this paper:

CAS Gramene: AT5G23060
CAS Araport: AT5G23060

KEA3 Gramene: AT4G04850
KEA3 Araport: AT4G04850

PGR5 Gramene: AT2G05620
PGR5 Araport: AT2G05620

STN8 Gramene: AT5G01920
STN8 Araport: AT5G01920

application CHEBI: CHEBI:33232

npq4 Gramene: AT1G44575
npq4 Araport: AT1G44575

NADPH CHEBI: CHEBI:16474
