# Supplementary material for: Diatoms exhibit dynamic chloroplast calcium signals in response to high light and oxidative stress
Source: Plant Physiol. 2024 Nov 9;197(1):kiae591. doi: 10.1093/plphys/kiae591 (PMC11663583; doi:10.1093/plphys/kiae591)
Supplement: kiae591_Supplementary_Data [file kiae591_supplementary_data.pdf]

Supplementary Data

Supplementary Figure S1: Relative fluorescence levels in Ca^{2+} reporter lines.

Supplementary Figure S2: Excitation protocols used during Ca^{2+} imaging.

Supplementary Figure S3: External Ca^{2+} is not required for the high light induced $[Ca^{2+}]_{str}$ elevations.

Supplementary Figure S4: Simultaneous measurement of Ca^{2+} in the cytosol and chloroplast

Supplementary Figure S5: Spatial specificity in $[Ca^{2+}]_{str}$ chl elevations.

Supplementary Figure S6: Characterization of the G-GECO1-mApple reporter.

Supplementary Figure S7: Sustained $[Ca^{2+}]_{str}$ elevations after continuous light stress.

Supplementary Figure S8: NH_4Cl induces $[Ca^{2+}]_{str}$ elevations.

Supplementary Figure S9: Longer term impacts of exogenous H_2O_2 on growth and photophysiology.

Supplementary Figure S10: Calibration of roGFP2-Orp1 for single cell microscopy.

Supplementary Figure S11: Effect of high light on Ca^{2+} and H_2O_2 in the cytosol.

Supplementary Figure S12: Effect of DCMU on Ca^{2+} and H_2O_2 in the chloroplast.

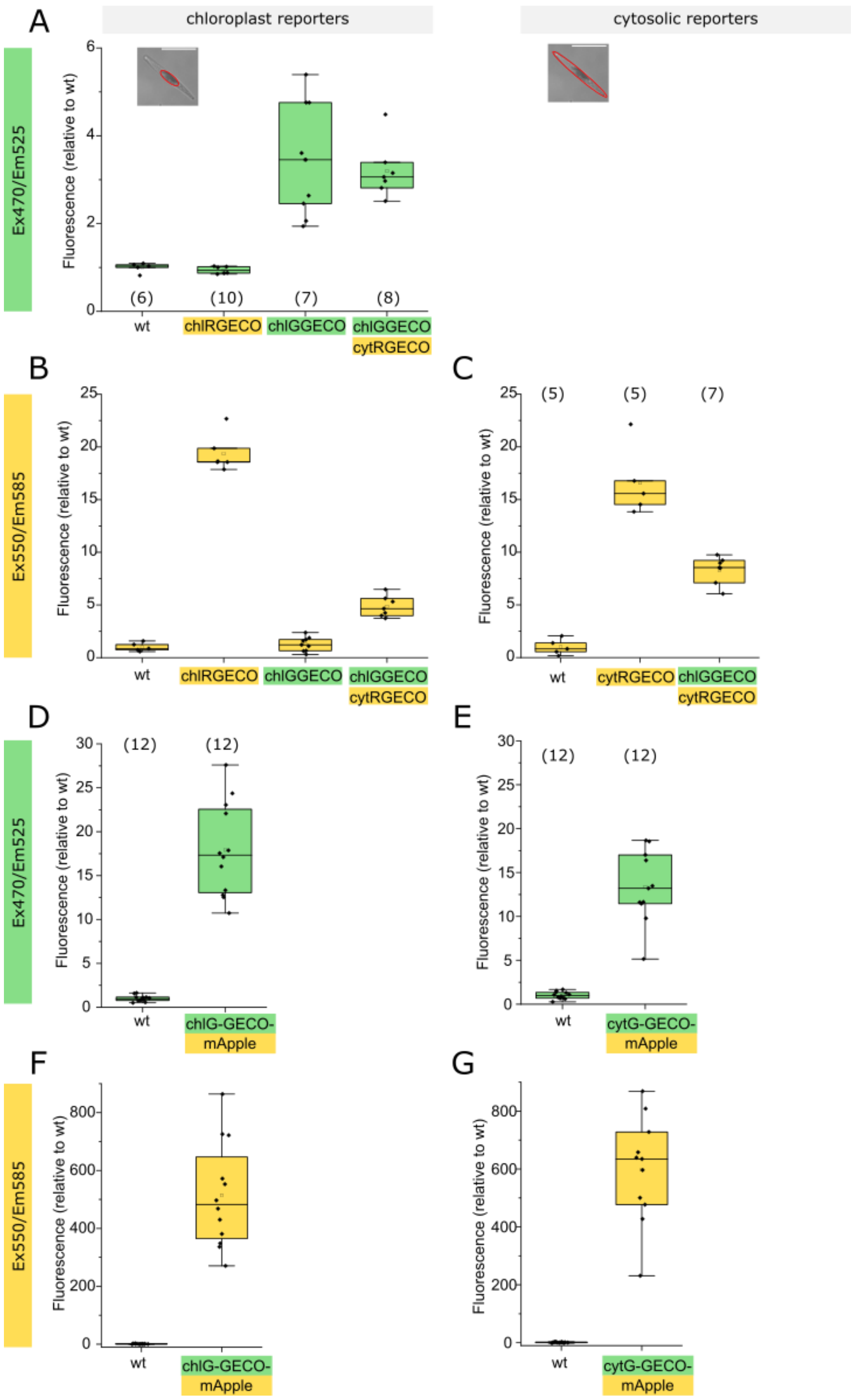

Supplementary Figure S1: Relative fluorescence levels in Ca^{2+} reporter lines. A) Resting fluorescence in chloroplast-localised reporter lines relative to wild type when excited at 470 nm, emission 525 nm using a region of interest (ROI) around the chloroplast. Mean resting fluorescence in chl-G-GECO1 is 3.5 fold greater than of wild-type. The box plot indicates

interquartile range (IQR) (25-75%), whiskers 1.5 IQR. The median (line) and mean (open square) are also shown. The same parameters were used for all box plots in this figure. Inset shows position of ROI. Scale bar = 10 µm. **B)** Resting fluorescence in chloroplast-localised reporter lines relative to wild type when excited at 550 nm, emission 585 nm. Mean resting fluorescence in chl-R-GECO1 is 19.3 fold greater than of wild-type. The dual reporter strain (chl-G-GECO1 and cyt-R-GECO1) shows elevated fluorescence due to the presence of cytosolic R-GECO1 in the vicinity of the chloroplast. **C)** Resting fluorescence in cytosol localised reporter lines relative to wild type when excited at 550 nm, emission 585 nm using a region of interest (ROI) around the entire cell. Mean resting fluorescence in cyt-R-GECO1 is 16.6 fold greater than of wild-type. **D)** Mean resting fluorescence of G-GECO1 in chl-G-GECO1-mApple is 17.9 fold greater than of wild-type (ex 470 nm, em 525 nm). **E)** Mean resting fluorescence of G-GECO1 in cyt-G-GECO1-mApple is 13.4 fold greater than wild-type (ex 470 nm, em 525 nm). **F)** Relative fluorescence of mApple in chl-G-GECO1-mApple relative to wild type (ex 550 nm, em 585 nm). **G)** Relative fluorescence of mApple in cyt-G-GECO1-mApple reporter lines relative to wild type (ex 550 nm, em 585 nm).

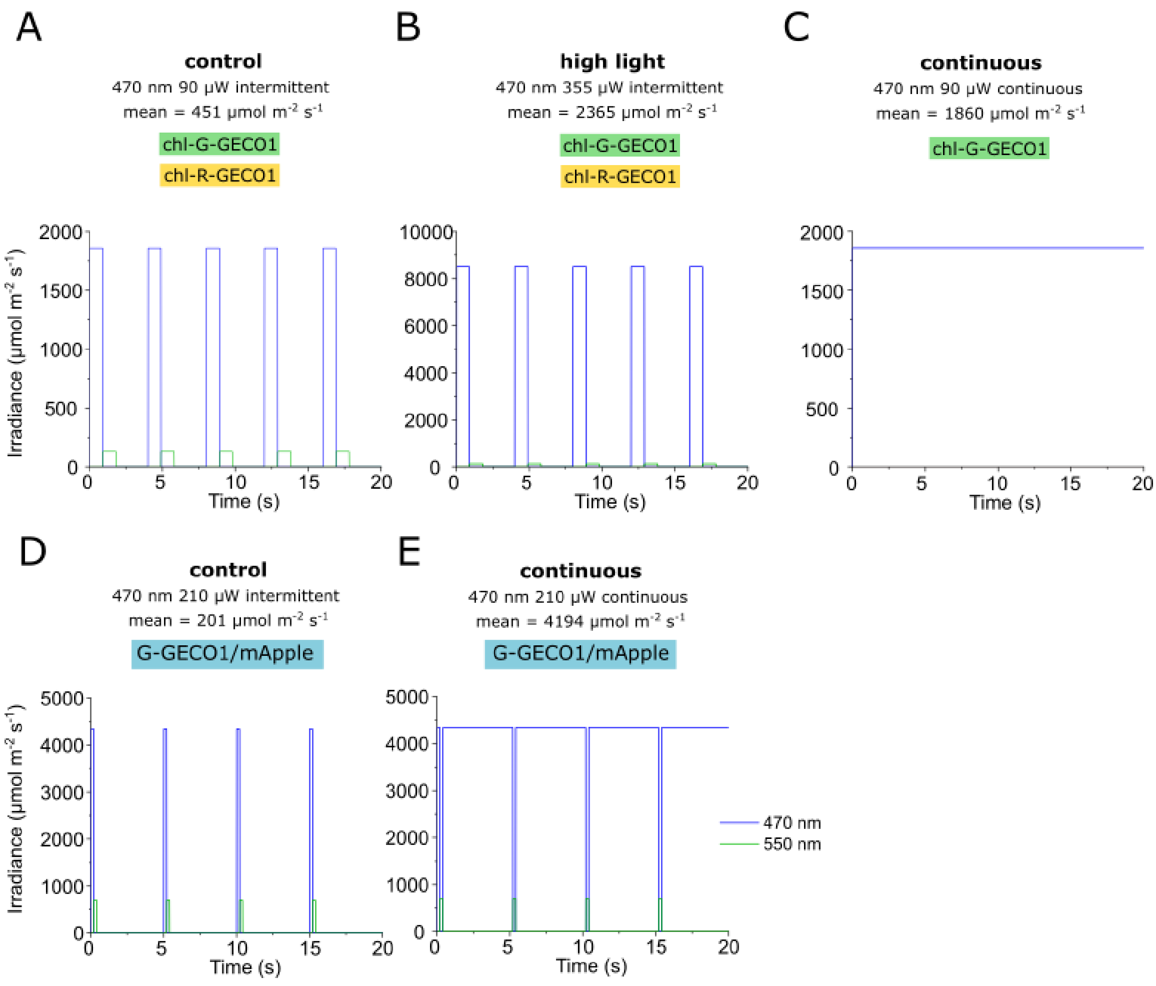

Supplementary Figure S2: Excitation protocols used during Ca^{2+} imaging. A schematic view of the irradiance experienced during different imaging protocols. Different illumination systems were used for single wavelength (A-C, chl-R-GECO1 and chl-G-GCEO-1) and ratiometric (D-E, chl-G-GECO1-mApple) indicators. **A)** Control imaging conditions for single wavelength indicators, intermittent illumination every 4 s. The mean irradiance (470 +550 nm) experienced by the cells is shown. Note that dual excitation (470 and 550 nm) was applied to all cells regardless of fluorophore to ensure that exposure to light was identical between indicators. **B)** High light imaging, as (A) but with a higher intensity for the 470 nm LED. Note the vertical axis is different. **C)** Continuous light, 470 nm LED at standard intensity. No 550 nm excitation was used. **D)** Control imaging conditions for the ratiometric indicator G-GECO1-mApple, intermittent illumination every 5 s. **E)** Continuous light from 470 nm LED at standard intensity, switching briefly to 550 nm for 0.2 s every 5 s.

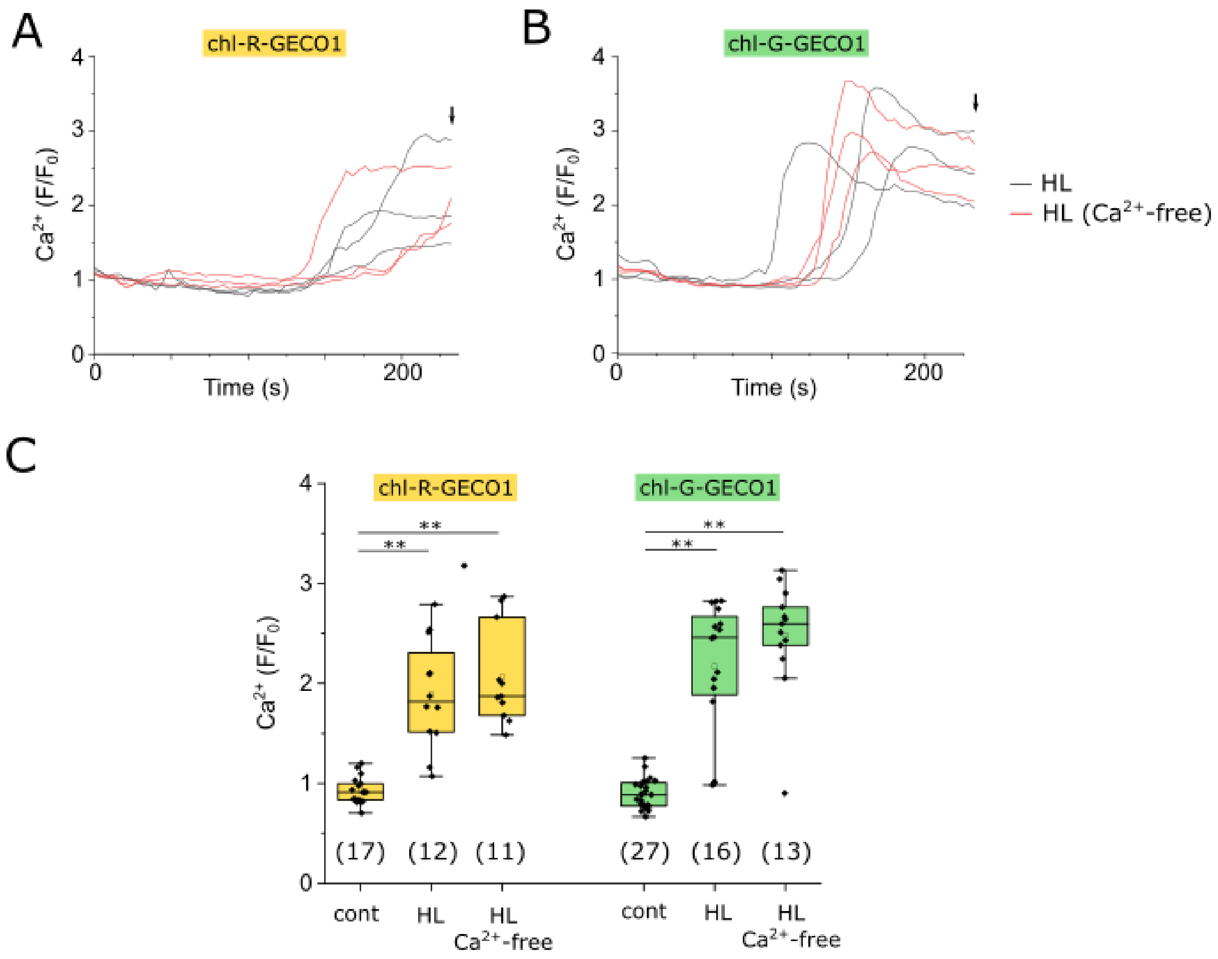

Supplementary Figure S3: External Ca^{2+} is not required for the high light induced $[Ca^{2+}]_{str}$ elevations. A) chl-R-GECO1 cells were resuspended in artificial seawater (ASW) containing either 10 mM Ca^{2+} (control) or 0 mM Ca^{2+} + 200 µM EGTA (Ca^{2+} free). Cells were imaged using high light (HL) conditions (intermittent excitation every 4 s using higher intensity 470 nm, mean irradiance 2365 µmol m^{-2} s^{-1}) to promote sustained $[Ca^{2+}]_{str}$ elevations. Three representative traces are shown for each treatment, total cell numbers examined is shown in (c). **B)** As in (A) but using chl-G-GECO1 cells. Three representative traces are shown for each treatment, total cell numbers examined is shown in (c). **C)** The box plot shows the change in $[Ca^{2+}]_{str}$ (F/F_0) after 240 s. Removing external Ca^{2+} does not inhibit the $[Ca^{2+}]_{str}$ elevations caused by high light. ** = $p<0.01$ ANOVA with Tukey post-hoc. The box plot indicates interquartile range (IQR) (25-75%), whiskers 1.5 IQR. The median (line) and mean (open square) are also shown.

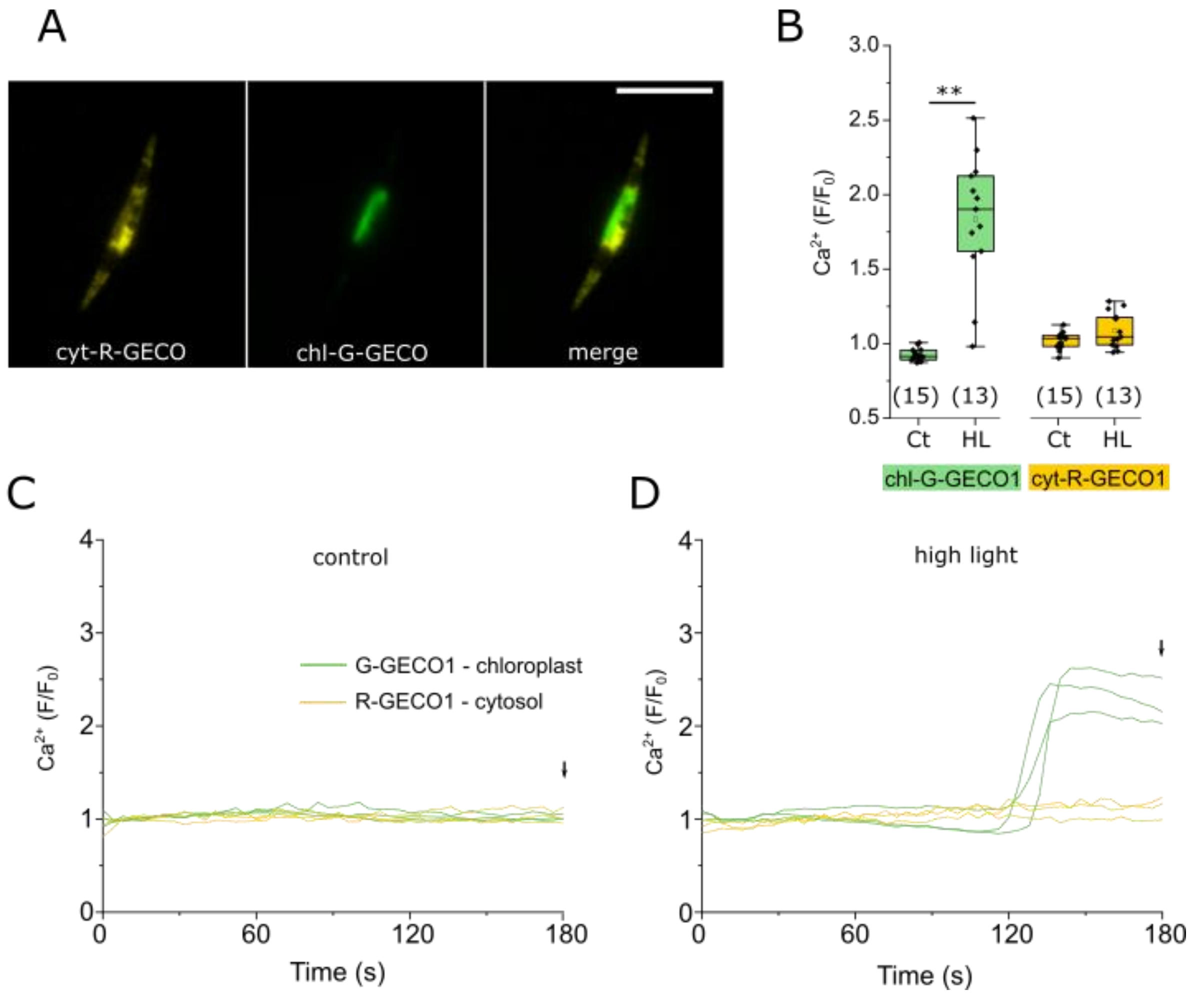

Supplementary Figure S4: Simultaneous measurement of Ca^{2+} in the cytosol and chloroplast. A) An epifluorescent image of a *P. tricornutum* strain expressing dual Ca^{2+} reporters, G-GECO1 in the chloroplast and R-GECO1 in the cytosol. Bar = 5 µm. **B)** Cells were imaged under control (Ct) and high light (HL) conditions (intermittent excitation every 4 s at 470 nm, mean irradiance 2365 µmol m^{-2} s^{-1}). These conditions cause a substantial rise in $[Ca^{2+}]_{str}$, but $[Ca^{2+}]_{cyt}$ is not affected by this increase. The box plot shows the change in fluorescence from initial (F/F_0) after 180 s. n= 15 control, n=13 high light. ** = p<0.01 ANOVA with Tukey post-hoc. The box plot indicates interquartile range (IQR) (25-75%), whiskers 1.5 IQR. The median (line) and mean (open square) are also shown. **C)** Representative traces from the data shown in (B) from cells under control conditions (n=3 traces shown). **D)** Representative traces from the data shown in (B) from cells under high light conditions (traces from 3 cells shown).

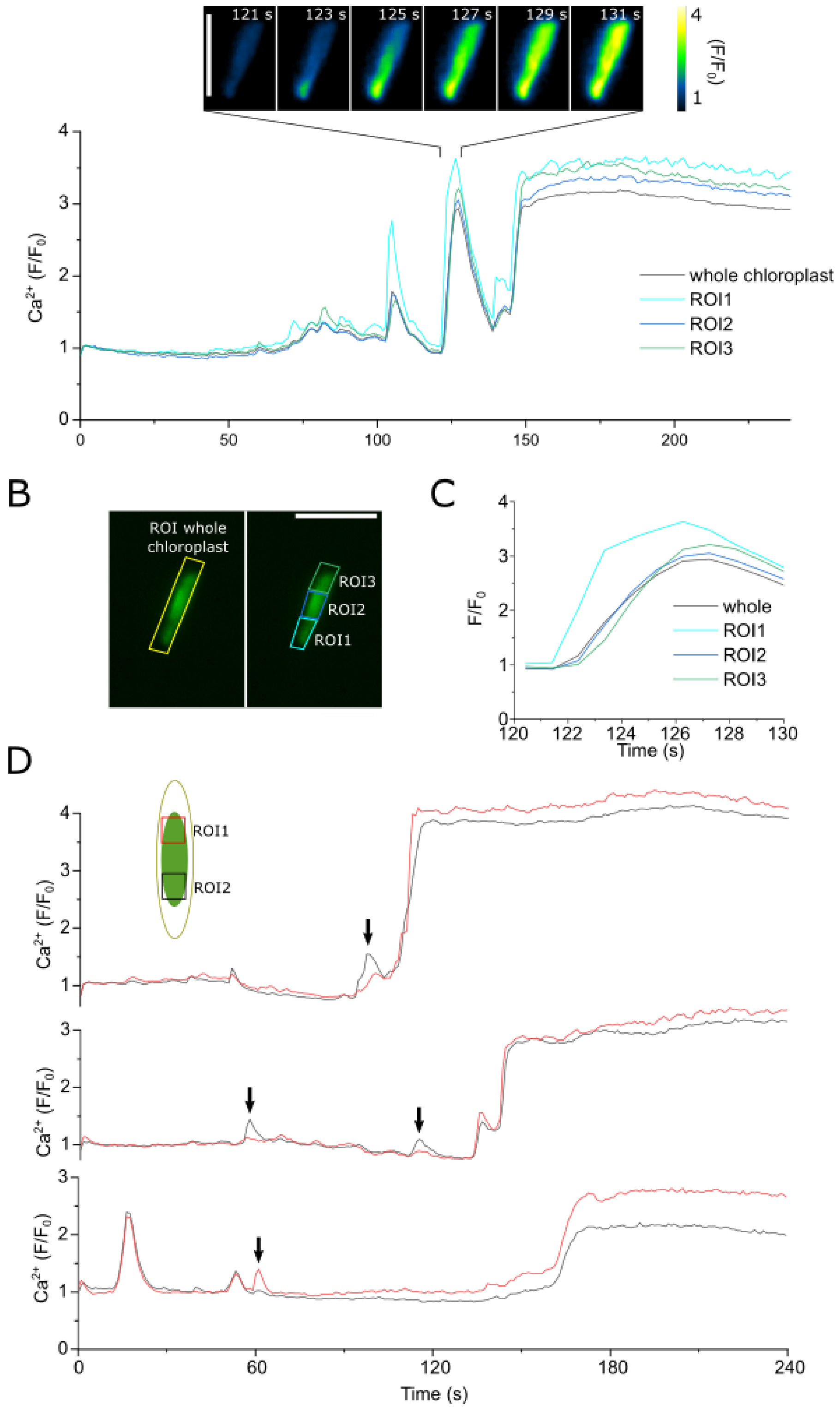

Supplementary Figure S5: Spatial specificity in $[Ca^{2+}]_{str}$ elevations. A) $[Ca^{2+}]_{str}$ elevations measured in chl-G-GECO1 cells induced by continuous illumination (continuous 470 nm illumination at standard intensity, mean irradiance 1860 µmol m^{-2} s^{-1}). Four regions of interest (ROI) were examined, encompassing three individual regions within the chloroplast area, and a ROI for the whole chloroplast. Distinct differences in the timing and amplitude of $[Ca^{2+}]_{str}$

elevations can be observed in the individual ROIs. False colour images showing relative change in fluorescence (F/F_0) demonstrate the localised initiation of the $[Ca^{2+}]_{str}$ elevation at 120 s. Bar = 5 µm. **B)** Epifluorescent microscopy image showing absolute fluorescence of the chloroplast examined in (A) and the position of the ROIs. Bar = 5 µm. **C)** Expanded view of the $[Ca^{2+}]_{str}$ elevation at 120 s showing an early onset in ROI1, followed by a later rise in the other ROIs. **D)** Further examples of localised $[Ca^{2+}]_{str}$ elevations in chl-G-GECO1 cells exposed to continuous light. Two ROIs were analysed at opposing ends of the chloroplast. ROI1 (red) and ROI2 (black). Whilst most $[Ca^{2+}]_{str}$ elevations are seen in both ROIs, each cell also shows localised $[Ca^{2+}]_{str}$ elevations (arrowed). Three cells are shown.

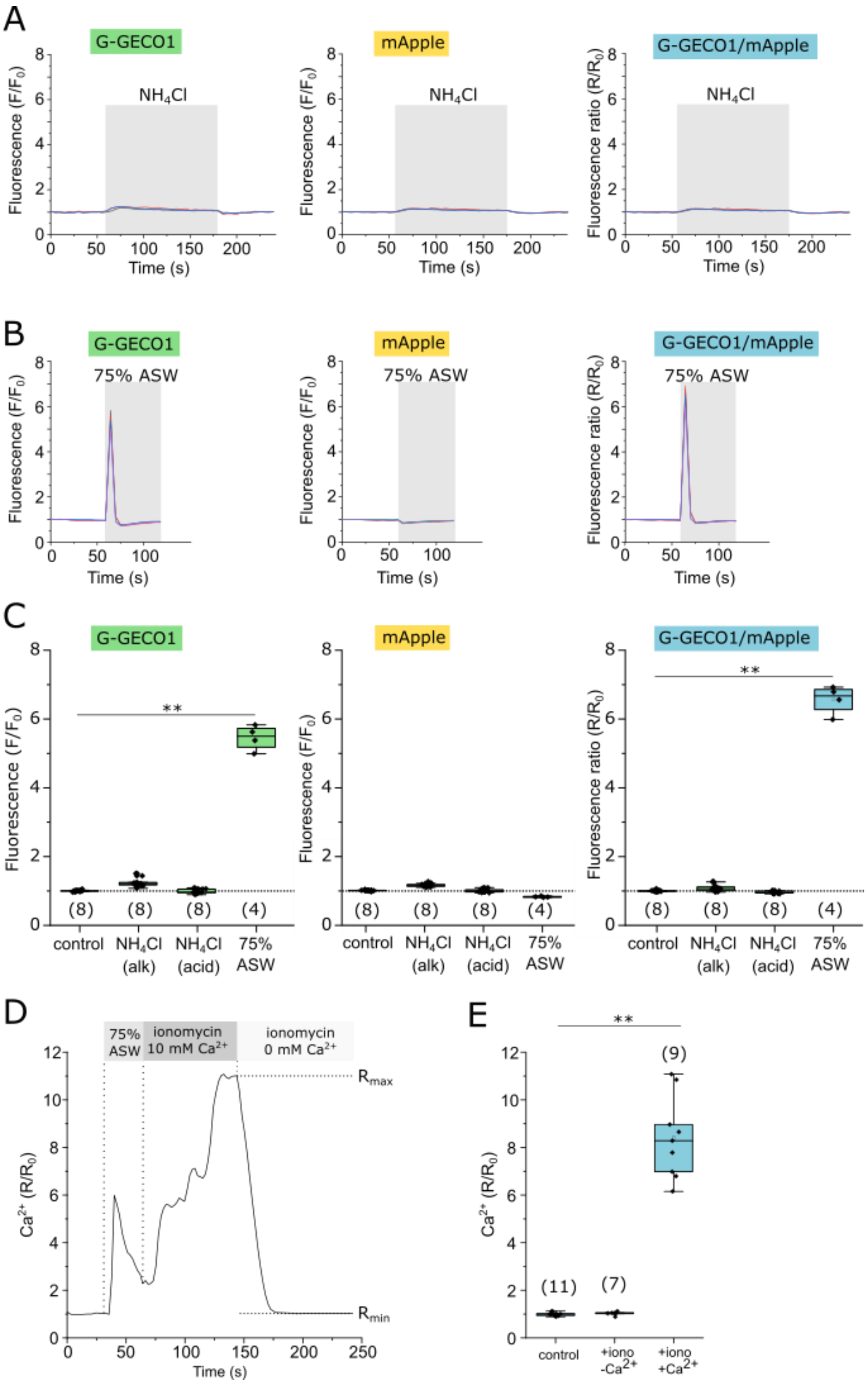

Supplementary Figure S6: Characterization of the cytosolic G-GECO1-mApple reporter. A) Impact of changes in cytosolic pH on cytosol-localised G-GECO1-mApple. The cells were perfused with 10 mM NH_4Cl, which induces strong cytosolic alkalinization, followed by a transient cytosolic acidification when it is removed. Both G-GECO1 and mApple exhibit a low sensitivity to pH, which is further reduced when the sensor is used ratiometrically (G-GECO1/mApple). 4 representative traces are shown, total cell numbers examined are shown

in (c). Note that external Ca^{2+} was not removed in these perfusions, although we did not observe $[Ca^{2+}]_{cyt}$ elevations. **B)** The response of G-GECO1-mApple to $[Ca^{2+}]_{cyt}$ elevations induced by hypo-osmotic shock. The cells were exposed to 75% artificial seawater (ASW), which induces a large increase in G-GECO1 fluorescence, but not mApple. 4 representative traces are shown, total cell numbers examined are shown in (c). **C)** Box plots demonstrating the different sensitivity of G-GECO1 and mApple to changes in pH_{cyt} and $[Ca^{2+}]_{cyt}$. The mean increase in R/R_0 in response to alkalinisation was 1.25±0.1 whereas the mean increase in response to hypo-osmotic shock was 6.56±0.4 (±sd). n=8, 8 and 4 cells for control, NH_4Cl and 75% ASW respectively. The box plot indicates interquartile range (IQR) (25-75%), whiskers 1.5 IQR. The median (line) and mean (open square) are also shown. **D)** Calibration of cyt-G-GECO1-mApple using the Ca^{2+} ionophore ionomycin. Cells were perfused with 75% ASW to induce a hypo-osmotic shock, followed by ASW containing 50 µM ionomycin with 10 mM Ca^{2+} (to estimate R_{max}) and then ASW containing 50 µM ionomycin with 0 mM Ca^{2+} and 500 µM EGTA (to estimate R_{min}). **E)** Box plots showing estimation of R_{max} and R_{min} following treatment with 50 µM ionomycin. The box plot indicates interquartile range (IQR) (25-75%), whiskers 1.5 IQR. The median (line) and mean (open square) are also shown.

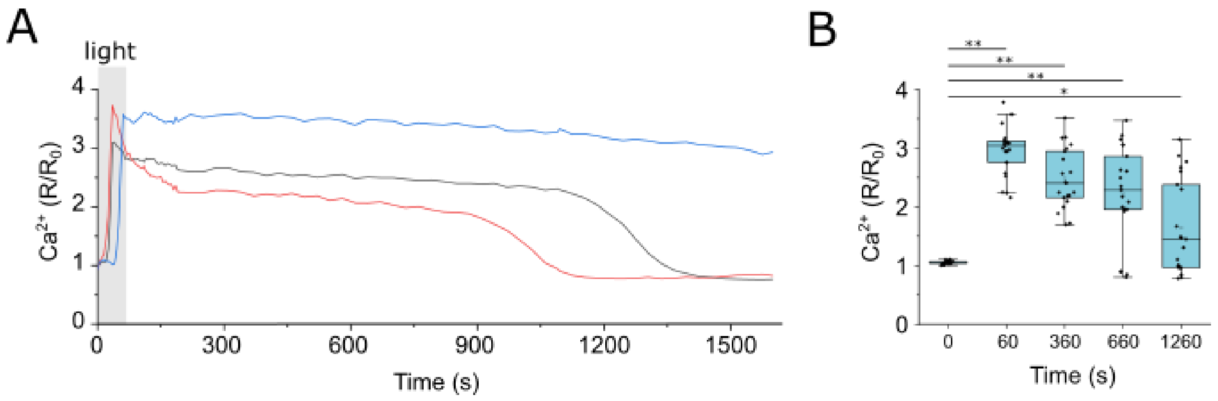

Supplementary Figure S7: Sustained $[Ca^{2+}]_{str}$ elevations after continuous light stress. A) Cells expressing chl-G-GECO1-mApple were exposed to continuous light (continuous 470 nm light, mean irradiance 4194 µmol m^{-2} s^{-1}) for 1 minute (between 0-60 s) to induce $[Ca^{2+}]_{str}$ elevations. The continuous light stress was removed and the cells were monitored over 25 minutes to examine the duration of the $[Ca^{2+}]_{str}$ elevation (imaging every 5 s between 60-240 s and every 30 s thereafter). All cells showed sustained $[Ca^{2+}]_{str}$ elevations. In 13 out of 21 cells, $[Ca^{2+}]_{str}$ returned to resting values within the 25 minute period. Note that the return to resting values was not gradual, but occurred within a 1-2 minute period. Three representative traces are shown, a different colour representing each cell (n=21 total cells examined). **B)** Changes in $[Ca^{2+}]_{str}$ during the recovery from the continuous light stress described in (A). n=21 cells. ** = $p<0.01$, * = $p<0.05$, means are significantly different from initial. ANOVA with Tukey post-hoc. The box plot indicates interquartile range (IQR) (25-75%), whiskers 1.5 IQR. The median (line) and mean (open square) are also shown.

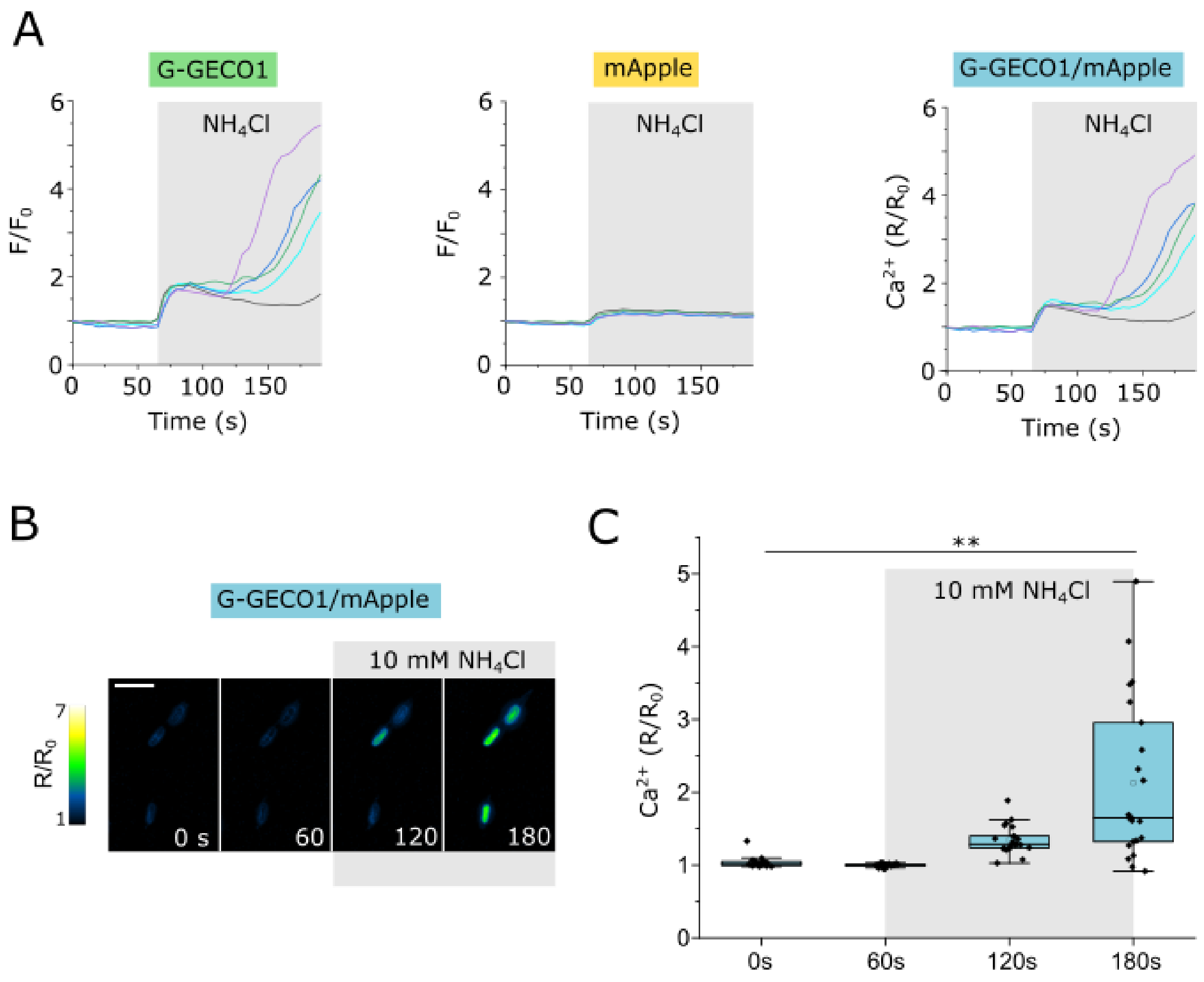

Supplementary Figure S8: NH_4Cl induces $[Ca^{2+}]_{str}$ elevations. A) Representative traces from individual chl-G-GECO1-mApple cells treated with 10 mM NH_4Cl. The traces indicate changes in G-GECO1 and mApple fluorescence, along with the normalized ratio. Addition of 10 mM NH_4Cl at 60 s results in an immediate small increase in the G-GECO-mApple ratio. This increase is most likely caused by alkalization of the stroma as it is observed in both the G-GECO1 and mApple traces. 75% of cells exhibited a subsequent large sustained increase in the G-GECO-mApple ratio that initiated after 120 s. This increase was observed solely in the G-

GECO1 trace indicating that it is caused by a change in $[Ca^{2+}]_{str}$. **B)** False colour images demonstrating large increases in R/R_0 after addition of NH_4Cl for 2 minutes. Bar=5 µm **C)** $[Ca^{2+}]_{str}$ elevations following addition of 10 mM NH_4Cl. The $[Ca^{2+}]_{str}$ was not significantly different from initial after 120 s (mean R/R_0 1.35±0.19) but was significantly different after 180 s (mean R/R_0 2.13±1.11). ** = $p<0.01$ (one-way ANOVA, Tukey post-hoc). n=20 cells. The box plot indicates interquartile range (IQR) (25-75%), whiskers 1.5 IQR. The median (line) and mean (open square) are also shown.

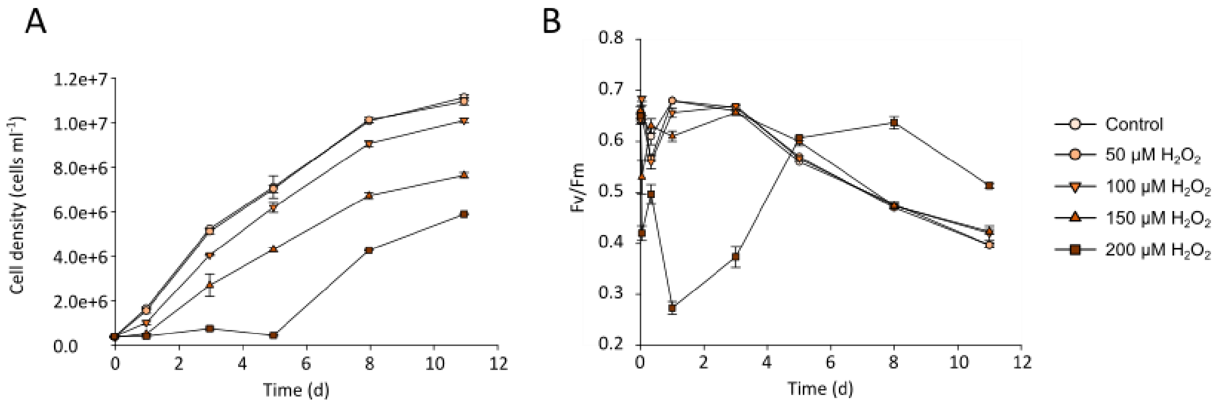

Supplementary Figure S9: Longer term impacts of exogenous H_2O_2 on growth and photophysiology. A) Growth rates of cyt-roGFP2-Orp1 cells treated with H_2O_2 at time 0 d. H_2O_2 was added to growth media at the start of the experiment. In all H_2O_2 treatments except 50 μM, growth is significantly impaired after 1 d, indicating that treatment with H_2O_2 has longer-term impacts on physiology. n=8. Error bars represent sd. **B)** Photosynthetic efficiency of PSII (F_v/F_m) for the experiment described in (A). All H_2O_2 treatments lead to a short-term decline in F_v/F_m that recovers within 24 h, except for 200 μM H_2O_2 where recovery is much slower. All treatments including the control show a gradual decline of F_v/F_m as cultures approach stationary phase. n=3. Error bars represent sd.

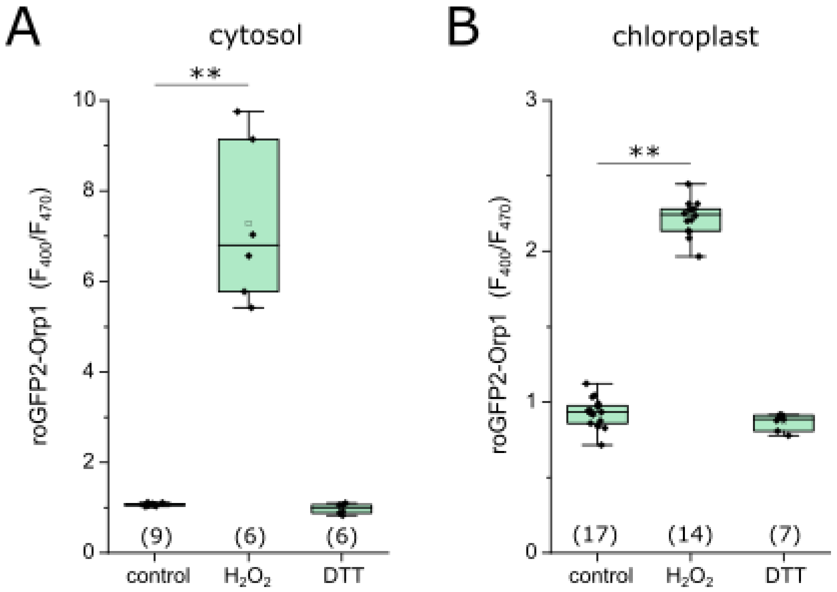

Supplementary Figure S10: Calibration of roGFP2-Orp1 for single cell microscopy. A) Determination of cyt-roGFP2-Orp1 fluorescence ratio (F_{400}/F_{470}) when fully oxidised (1 mM H_2O_2) or fully reduced (1 mM DTT). Measurements were taken using epifluorescent microscopy after treatment had been applied for 3 minutes. ** = mean is significantly different from untreated control, p<0.01 (one-way ANOVA, Tukey post-hoc). The box plot indicates interquartile range (IQR) (25-75%), whiskers 1.5 IQR. The median (line) and mean (open square) are also shown. **B)** Single cell determination of chl-roGFP2-Orp1 fluorescence ratio after treatment with 1 mM H_2O_2 or 1 mM DTT for 3 minutes. Note that the dynamic range of the chloroplast localised reporter differs from the cytosol. ** = mean is significantly different from untreated control, p<0.01 (one-way ANOVA, Tukey post-hoc). The box plot indicates interquartile range (IQR) (25-75%), whiskers 1.5 IQR. The median (line) and mean (open square) are also shown.

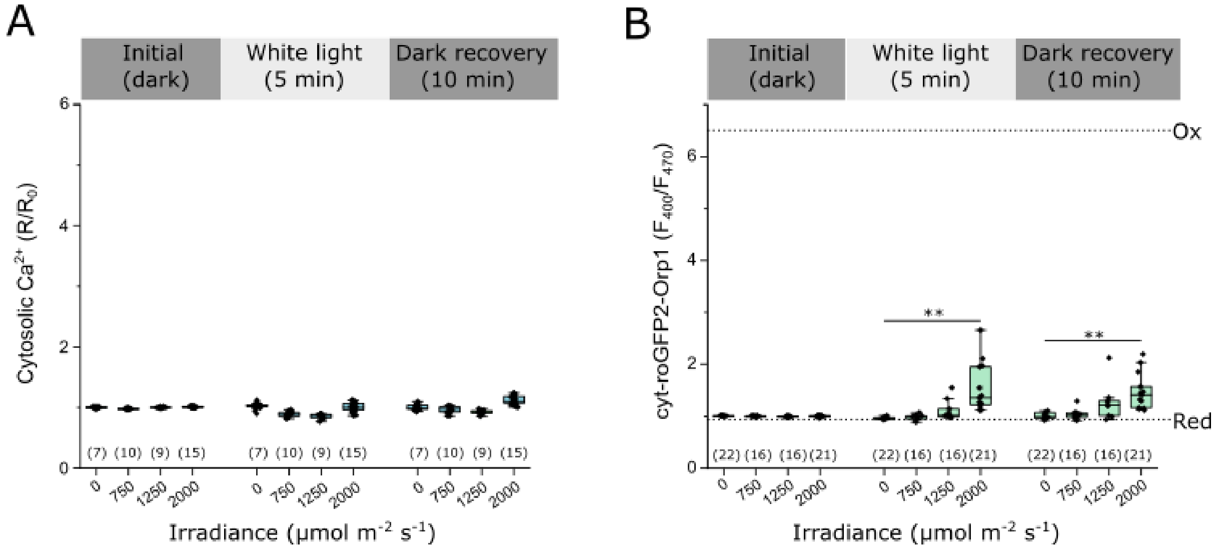

Supplementary Figure S11: Effect of high light on Ca^{2+} and H_2O_2 in the cytosol. A) Changes in $[Ca^{2+}]_{cyt}$ following exposure of cyt-G-GECO-mApple cells to continuous white light (0, 750, 1250, 2000 µmol m^{-2} s^{-1}). The change in $[Ca^{2+}]_{cyt}$ is shown before treatment, immediately after exposure to light (5 minutes) and then after 10 minutes of dark recovery. No treatments were significantly different from the control ($p>0.05$, one-way ANOVA, Tukey post-hoc). The box plot indicates interquartile range (IQR) (25-75%), whiskers 1.5 IQR. The median (line) and mean (open square) are also shown. **B)** Box plots showing changes in cytosolic H_2O_2 following exposure to 5 minutes of continuous white light (0, 750, 1250, 2000 µmol m^{-2} s^{-1}). Individual cyt-roGFP2-Orp1 cells were exposed to light as described in (A). Fluorescence ratio was determined before treatment, immediately after exposure to light (5 minutes) and then after 10 minutes dark recovery. ** = significantly different from control (dark treated) value, $p<0.01$ (one-way ANOVA, Tukey post-hoc). Dotted lines indicate F_{400}/F_{470} for fully oxidised (ox, 1 mM H_2O_2) and fully reduced probes (red, 1 mM dithiothreitol, DTT). The box plot indicates interquartile range (IQR) (25-75%), whiskers 1.5 IQR. The median (line) and mean (open square) are also shown.

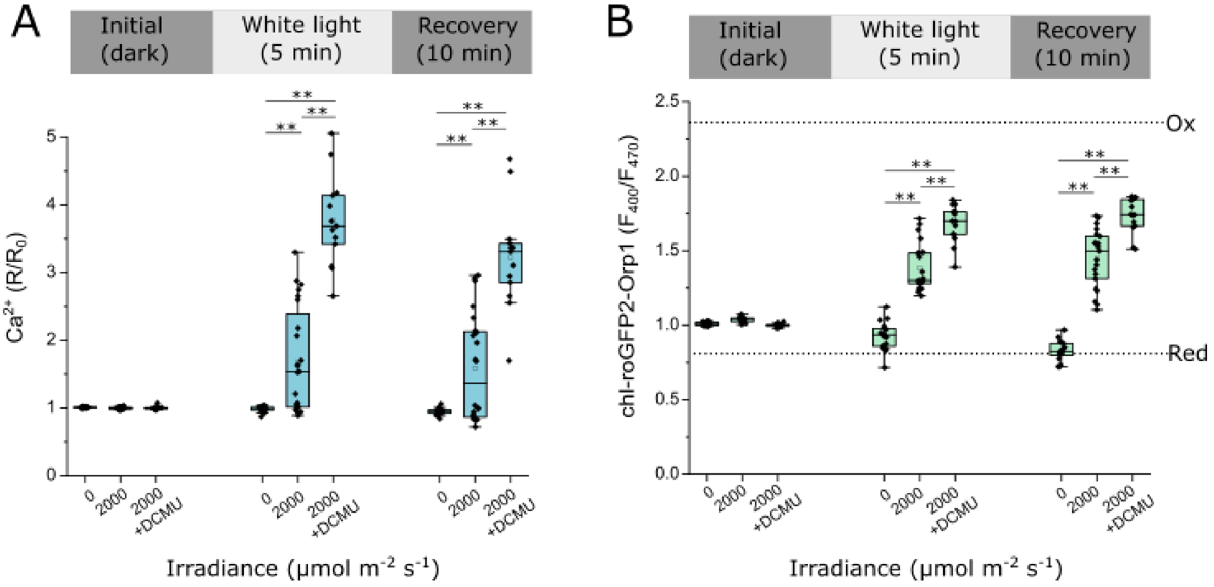

Supplementary Figure S12: Effect of DCMU on Ca^{2+} and H_2O_2 in the chloroplast. A) Changes in $[Ca^{2+}]_{str}$ following exposure of chl-G-GECO-mApple cells to continuous white light at 2000 µmol m^{-2} s^{-1}. Cells were pre-treated with 10 µM DCMU for ten minutes prior to the experiment. The change in $[Ca^{2+}]_{str}$ is shown before light, immediately after exposure to light (5 minutes) and then after 10 minutes of dark recovery. ** = significantly different means ($p<0.01$, one-way ANOVA, Tukey post-hoc). n = 18, 21 and 10 cells respectively. The box plot indicates interquartile range (IQR) (25-75%), whiskers 1.5 IQR. The median (line) and mean (open square) are also shown. **B)** Box plots showing changes in chlorplast H_2O_2 following treatment with 10 µM DCMU. Individual chl-roGFP2-Orp1 cells were exposed to light as described in (A). Fluorescence ratio was determined before treatment, immediately after exposure to light (5 minutes) and then after 10 minutes dark recovery. ** = significantly different, $p<0.01$ (one-way ANOVA, Tukey post-hoc). n = 17, 21 and 14 cells respectively. The box plot indicates interquartile range (IQR) (25-75%), whiskers 1.5 IQR. The median (line) and mean (open square) are also shown.
